# The Development of the Human Female Reproductive Tract. Part 1: Uterine Tube and Uterus

**DOI:** 10.1002/ca.70014

**Published:** 2025-10-06

**Authors:** Cindy J. M. Hülsman, S. Eleonore Köhler, Gabriela Morosan‐Puopolo, Jill P. J. M. Hikspoors, Wouter H. Lamers

**Affiliations:** ^1^ Department of Anatomy & Embryology Maastricht University Maastricht the Netherlands; ^2^ Department of Anatomy & Molecular Embryology Ruhr University Bochum Bochum Germany

**Keywords:** gubernaculum, mesonephric (Wolffian) duct, paramesonephric (Müllerian) duct, uterine tubes, uterus

## Abstract

The uterine tubes and uterus develop from the paramesonephric (Müllerian) ducts. Most experimental data are obtained in rodents. Since the (micro‐)anatomy of the murine urogenital tract differs from that in humans, evaluation of the translatability of mouse data to human development is relevant. We studied the Müllerian ducts in serially sectioned female human embryos and fetuses between 5 and 15 weeks of development and prepared 3D‐reconstructions to establish topographic relations. At 5 weeks of development, the dorsocranial peritoneal epithelium thickens locally to form a placode‐like structure, which remodels into the tubal orifice at 6 weeks. The subsequent caudal extension of the Müllerian ducts requires its temporary stay with the mesonephric (Wolffian) duct inside a common basement membrane. The site where the Müllerian segment expands passes as a wave along the Wolffian duct. This wave breaks when the tubes reach the lesser pelvis in the 8th week. There, both Müllerian ducts fuse to form the single uterovaginal canal. No fusion occurs most caudally, where the Müllerian ducts elicit the Müllerian tubercle in the dorsal wall of the urogenital sinus. The uterovaginal canal becomes encased in a mesenchymal cuff, the genital cord. The gubernaculum, which appears at 6.5 weeks as a tissue bridge between the mesonephros and the lateral body wall, eventually becomes the round ligament in females. At 12 weeks, it is still an intraperitoneal structure in an evagination of the abdominal cavity. Unexpectedly, the early development of the uterovaginal canal was similar in human and mouse embryos.

## 1 Introduction

The uterine tubes and uterus develop from the paramesonephric (Müllerian) ducts. The developmental biology of the uterine tubes probably represents the better‐studied feature of the Müllerian ducts. The tubes are easily accessible in the (embryonic) abdominal cavity, and the development of the uterine tubes in mammals and birds seems similar. The finding that the destruction of the Wolffian duct in the chick embryo prevents the extension of the caudal end of the Müllerian duct towards the cloaca (Grünwald 1937) had further shown that interventional studies are well tolerated by the system. The temporal absence of a basement membrane between the intimately close epithelial areas of the Wolffian and Müllerian ducts (Gruenwald 1941) further suggested an exchange of signaling molecules between the two organs. Based on similar experimental approaches, a molecular description of uterine‐tube development is presently becoming available [for recent reviews see (Roly et al. 2018; Santana Gonzalez et al. 2021; Machado et al. 2022)].

Nearly all of the recent studies on the development of the Müllerian ducts have been carried out in mice. Since the (micro‐)anatomy of the urogenital tract of rodents at first glance differs substantially from that in humans (Kurita 2010), an evaluation of the translatability of mouse data to human development is relevant. In this study, we pursued a histological and topographical analysis of the early human Müllerian ducts to follow their growth and differentiation. The first part of this study focuses on the development of the uterine tubes and the uterus, while the second part describes the early formation and differentiation of the vagina (Hülsman, Köhler, et al. 2025). For both studies, we have produced 3D reconstructions to allow for topographic descriptions of the important structures. In some of the reconstructions, we have carried out measurements to propose mechanisms that underlie morphogenesis.

## 2 Materials and Methods

### 2.1 Embryos and Fetuses

This study used historical collections of anonymous human embryos and fetuses, which were compiled > 50 years ago. Such collections are exempt from ethical approval according to the Dutch regulations for the proper use of human tissue for (bio‐)medical research purposes. The study used digitized images of serial sections of anonymized specimens from the historical collections of the Departments of Anatomy and Embryology of Leiden University Medical Centre (LUMC), Leiden, Amsterdam University Medical Centers (AUMC), Amsterdam, Radboud University Medical Centre, Nijmegen, The Netherlands, and the Departments of Anatomy and Embryology of University Medical Centre, Georg‐August‐Universität, Göttingen (Blechschmidt Collection), and Ruhr University Bochum (Hinrichsen Collection), Germany (Table 1). Furthermore, embryos of the Carnegie Collection in Washington (DC), which are freely accessible on the internet, were included. The criteria of O'Rahilly, as modified in 2010 (O'Rahilly and Müller 2010) were used to determine Carnegie Stages (CS) and ages of development. The age of the fetuses (> 8 weeks of development) was based on the comparison of their crown‐rump length (CRL) with a reference graph, the construction of which is described in (Hülsman, Gao, et al. 2025a). The possible error in the estimated developmental age of the specimens used to construct that reference graph is ~1 week for fetuses up to ~20 weeks of development. We have studied the development of the human uterine tube and uterus, and its environment, in detail in 21 embryos and fetuses between CS15 (~5 weeks of development) and ~15weeks of development (Table 1). Sections and partial reconstructions of other embryos were studied if necessary.

**TABLE 1:** Overview of human embryos and fetuses used in the study.

Stage^a^	Weeks^b^	Number	CRL^c^ (mm)	Plane	Source
CS15	5	1954‐09‐15	9	Sagittal	Göttingen
CS15	5	S721	9	Transverse	DREM
CS16	5.5	1952‐04‐16	10.6	Transverse	Göttingen
CS17	6	S6520	14.2	Transverse	DREM
CS18	6	S4430	14	Transverse	DREM
CS18	6	S97	16	Transverse	LUMC
CS19	6.5	1949‐06_22	17.5	Sagittal	Göttingen
CS20	6.5	1947_07_02	16	Transverse	Göttingen
CS20	7	S2025	20	Transverse	AMC
CS21	7	S4090	22	Transverse	DREM
CS22	7.5	S983	28	Transverse	DREM
CS23	8	S48	40	Transverse	LUMC
CS23	8	S4141	35	Transverse	AMC
CS23	8	S88	24	Sagittal	Nijmegen
CS23	8	S9226	31	Transverse	DREM
	8.5	EYO295	32	Sagittal	Bochum
	9	S89	37	Transverse	LUMC
	10	S4908	50	Transverse	AMC
	10	S1744	60	Transverse	LUMC
	11	S1743	80	Sagittal	LUMC
	12	S2383	105	Transverse	LUMC
	13	S2212	110	Sagittal	LUMC
	15	S2392	130	Transverse	LUMC

^a^ Carnegie stage.

^b^ Week of development.

^c^ Crown‐Rump length.

### 2.2 Image Acquisition, 3D Reconstruction, and Visualization

Serial sections of human embryos from the Academic Medical Center (Amsterdam), Leiden University Medical Center, and Radboud Medical Center were digitized with an Olympus BX51 or BX61 microscope and the DOTSLIDE program (Olympus, Zoeterwoude, The Netherlands). The embryos from the University Medical Centre Göttingen and Ruhr University Bochum were scanned with a Zeiss Axioscan Z1 (Carl Zeiss Microscopy, Jena, Germany). Digital images of serial sections from the Carnegie collection were obtained via the Virtual Human Embryo Project (http://virtualhumanembryo.lsuhsc.edu). All images were converted into gray‐scale “JPEG” format and loaded into Amira3D (version 2020.2; base package; FEI Visualization Sciences Group Europe, Merignac Cedex, France). The gray‐scale images were aligned automatically with the least‐squares alignment mode and then manually adjusted for the correct curvature of the embryonic body axis with the help of photographs and magnetic resonance images of human embryos of the same stage (Pooh et al. 2011). Structures of interest were segmented manually and reconstructed three‐dimensionally with the Amira3D program. All epithelia were segmented following their basement membrane. Small deformations of individual sections due to the histological processing and stepwise stacking of sections conferred a distracting noise on the 3D reconstructions. Therefore, polygon meshes from all reconstructed materials were exported via “vrml export” from Amira3D to Cinema 4D (MAXON Computer GmbH, Friedrichsdorf, Germany) and remodeled using the Amira3D model as a template (Figure S1C). The accuracy of the remodeling process was safeguarded by simultaneous visualization in Cinema 4D of the original output from Amira3D and the remodeled Cinema model. Figure S1 shows a brief visual summary of the reconstruction procedure. The Cinema 4D models were transferred via “wrl export” to Adobe Acrobat version 9 (http://www.adobe.com) to generate interactive 3D Portable Device Format (PDF) files, which are an easily accessible format for 3D visualization.

### 2.3 Measurements

All length measurements have been performed by using the spline‐function in Cinema4D. To establish the segmental level of structures relative to the vertebral column, a line from the cranial rim of the symphysis to the structure of interest was extended to the vertebral column.

### 2.4 Three‐Dimensional Reconstructions Presented in PDF Format

In addition to the Figures, the evidence for our descriptions can be inspected in the corresponding interactive 3D‐PDFs (Figures [Link], [Link]). The reader is, therefore, encouraged to read the text and inspect the corresponding interactive PDFs simultaneously. This is because their rotational options (“live” images) allow a much better understanding of the complex local topography than do “still” images and text. The 3D‐PDFs can also be used to identify or verify the identity of a structure in a Figure: after opening and positioning the reconstruction similar to the view in the Figure, marking or unmarking a structure in the model tree will link the image to a name. Each of the stages of human development we studied is represented by a 3D‐PDF reconstruction. We do not refer to these 3D‐PDFs in the main text to avoid crowding the text with similar words.

## 3 Results

### 3.1 List of Abbreviations and Color Codes

**Table:** 

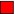	A	aorta
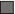	B	bladder
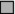	Br	bronchus
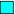	C	cervix
	Cau	caudal
	CCV	caudal cardinal veins
	Cr	cranial
	D	dorsal
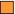	G	gubernaculum
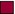	GC	genital cord
	H	hindgut (before 9 weeks)
	L	left
	Li	liver
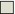	L5	lumbar vertebra 5
	M	mesonephros
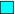	MD	Müllerian duct
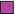	MDm	Müllerian duct mesenchyme
	Mo	mesovary
	Mu	muscles of abdominal wall
	Ov	ovary
	OL	proper ovarian ligament
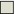	P	pubic bone
	PE	peritoneal cavity
	PL	pleural cavity
	R	right
	Re	rectum (after 9 weeks)
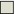	S	sacrum
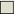	S1	sacral vertebra 1
	SL	suspensory ligament of ovary
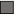	U	urethra
	Ut	uterus
	UA	umbilical artery
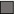	UGS	urogenital sinus
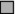	UP	urethral plate
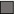	Ur	ureter
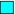	UVC	uterovaginal canal
	V	ventral
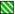	Va	vagina
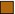	Vest	vaginal vestibule
	W	weeks
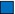	WD	Wolffian duct
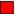	WDm	Wolffian duct mesenchyme

### 3.2 The Appearance and Early Development of the Müllerian Ducts

**FIGURE 1:**
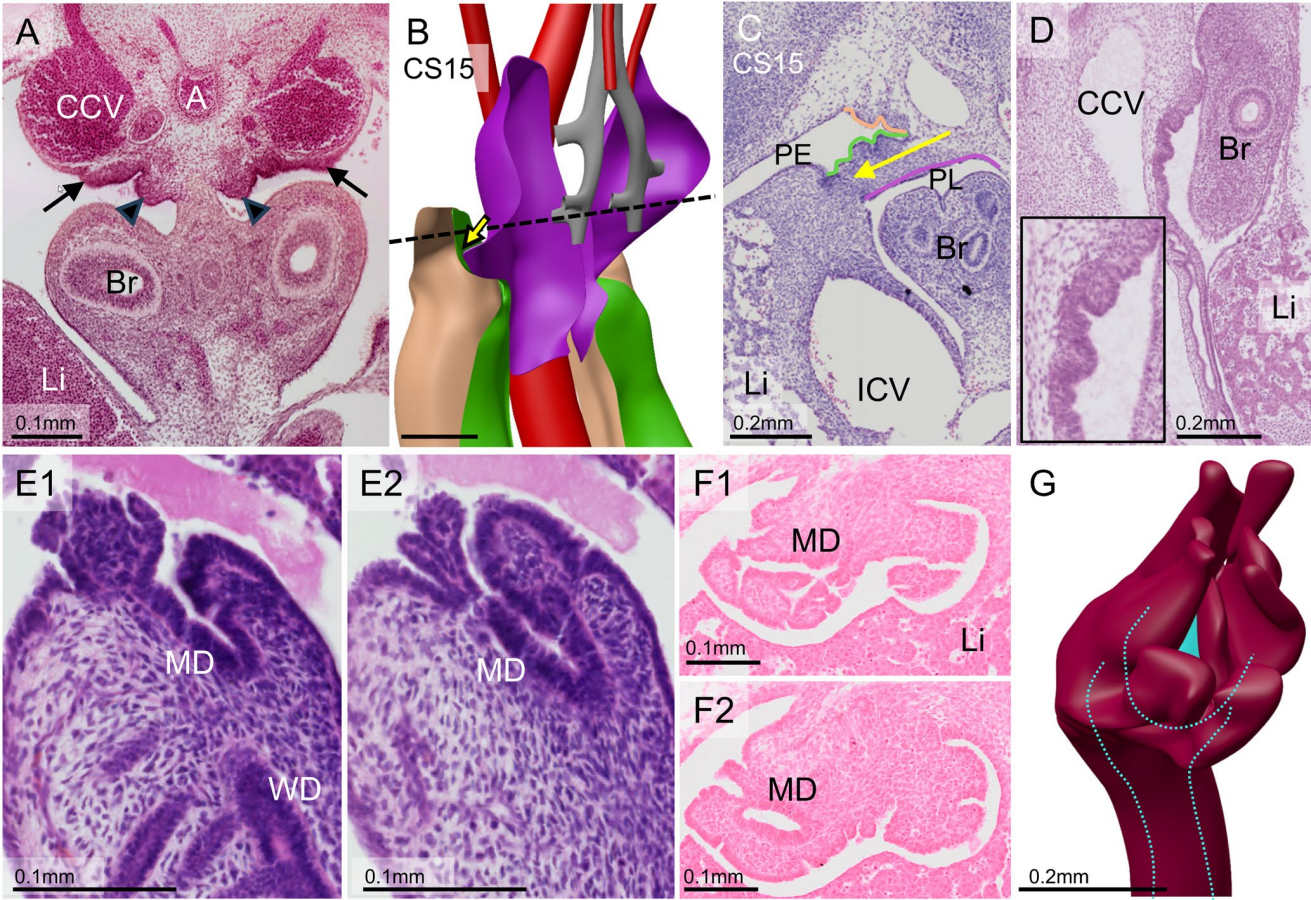
The early development of the Müllerian duct. Panels A–D show CS15 embryos (S721 and 1954‐09‐15) and panels E‐G CS18‐19 embryos (1949_06_22 and S97). Panel A is a transverse view of the bronchi of the lung and the (engorged) caudal cardinal veins. The arrows point at the Müllerian placode‐like structures and the arrowheads at the pleuroperitoneal membrane. Panel B is a left‐caudal view of the inferior surface of the parietal pleura (purple), the peritoneal surface of the pleuroperitoneal membrane (green), and the craniodorsal wall of the peritoneum (light orange). The bronchial tree is shown in gray and the dorsal aorta in red. The position of the pleuroperitoneal membrane is indicated by a yellow arrow in panel B (3D) and C (histology). Panel D shows the histology of a slightly older, sagittally sectioned embryo. Note in panels C and D that the surface of the pleuroperitoneal membrane is irregular on its peritoneal side, whereas the pleural side remains smooth. Panels E1 and E2 show serial sagittal sections shortly after the invagination of the infundibulum of the Müllerian duct has started. Panels F1 and F2 show the cranial part of the urogenital ridges, with the already existing Wolffian duct and the deepening furrows near the opening of the Müllerian duct. Panel G shows a reconstruction of the fimbria‐like structures at the entrance of the Müllerian duct. A: aorta; Br: bronchus; CCV: caudal cardinal veins; ICV: inferior caval vein; Li: liver; MD: Müllerian duct; PE: peritoneal cavity; PL: pleural cavity. Bars: 1 mm, unless stated otherwise.

**FIGURE 2:**
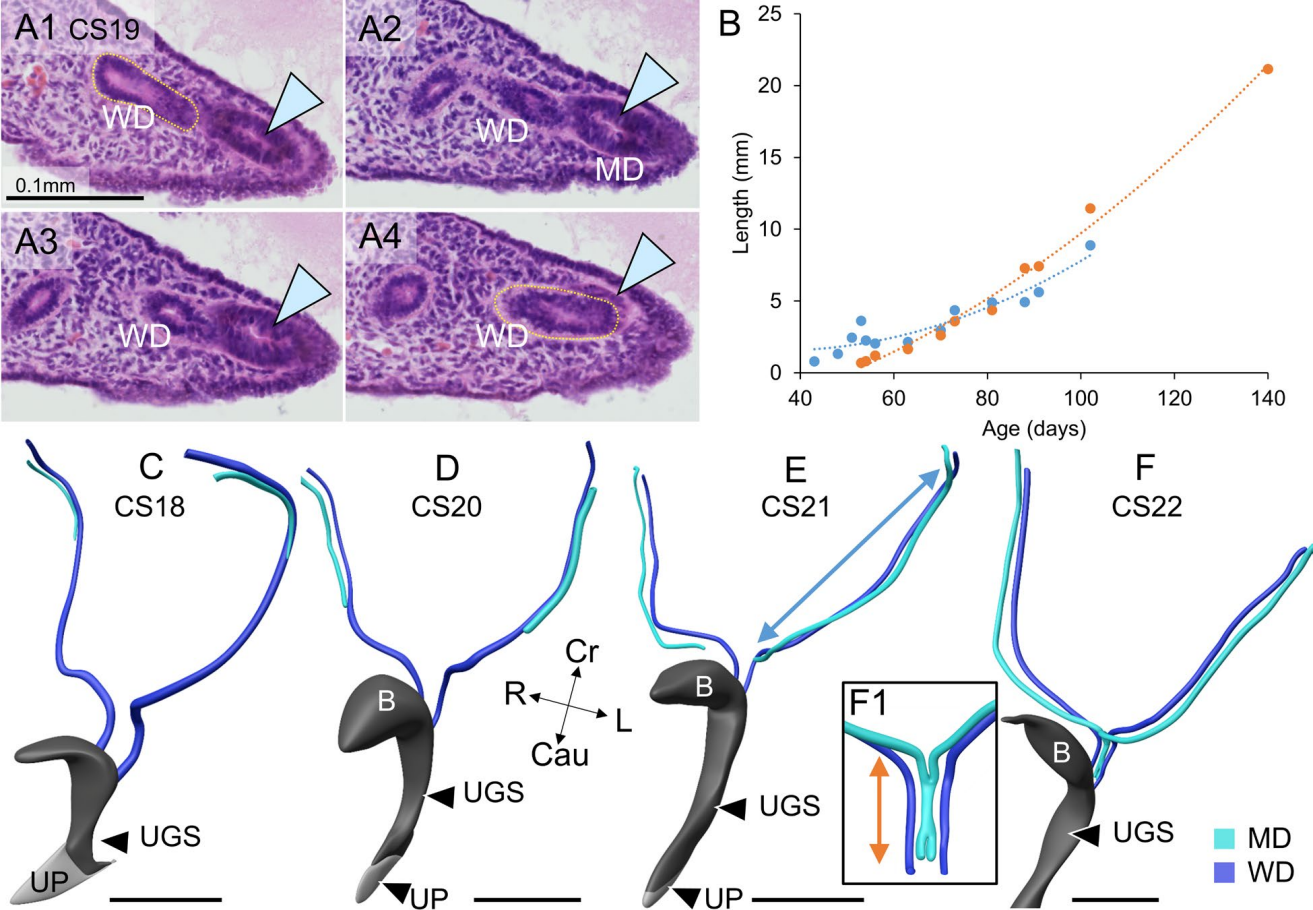
Caudal elongation of the Müllerian ducts and formation of uterovaginal canal. Panels A1–4 show quasi‐serial sections to demonstrate the close contact between the Müllerian and Wolffian ducts just prior to Müllerian duct elongation in caudal direction (CS19; 1949_06_22). The basement membrane of the Wolffian duct is highlighted for identification in panels A1 and A4. The common basement membrane is well visible in panels A2 and A3. Note the wedge shape of the tip of the Müllerian duct. Panel B shows the increase in length of the part of the Müllerian ducts that will become the uterine tubes (cyan double‐headed arrow in panel E) is relatively rapid in the stages shown in panels C–F, and then declines temporally. Growth in the uterovaginal canal is initially similar to that in the uterine tubes, but gradually declines thereafter (orange line). Note that the uterine tubes were not included in the 20‐week‐old fetal specimen. Panels C–F show reconstructions of the Wolffian ducts (dark blue), the still growing Müllerian ducts (cyan), and the corresponding urogenital sinuses (gray) between Carnegie stages 18 and 22 (S4430, S2025, S4090, and S983). Note the guiding function of the Wolffian ducts. In the CS22 embryo, the uterovaginal canal is forming (panel F1). B: bladder; Cau: Caudal; Cr: cranial; L: left; M: mesonephros; MD: Müllerian duct; R: right; UGS: urogenital sinus; UP: urethral plate; WD: Wolffian duct. Bars: 1 mm, unless stated otherwise.

**FIGURE 3:**
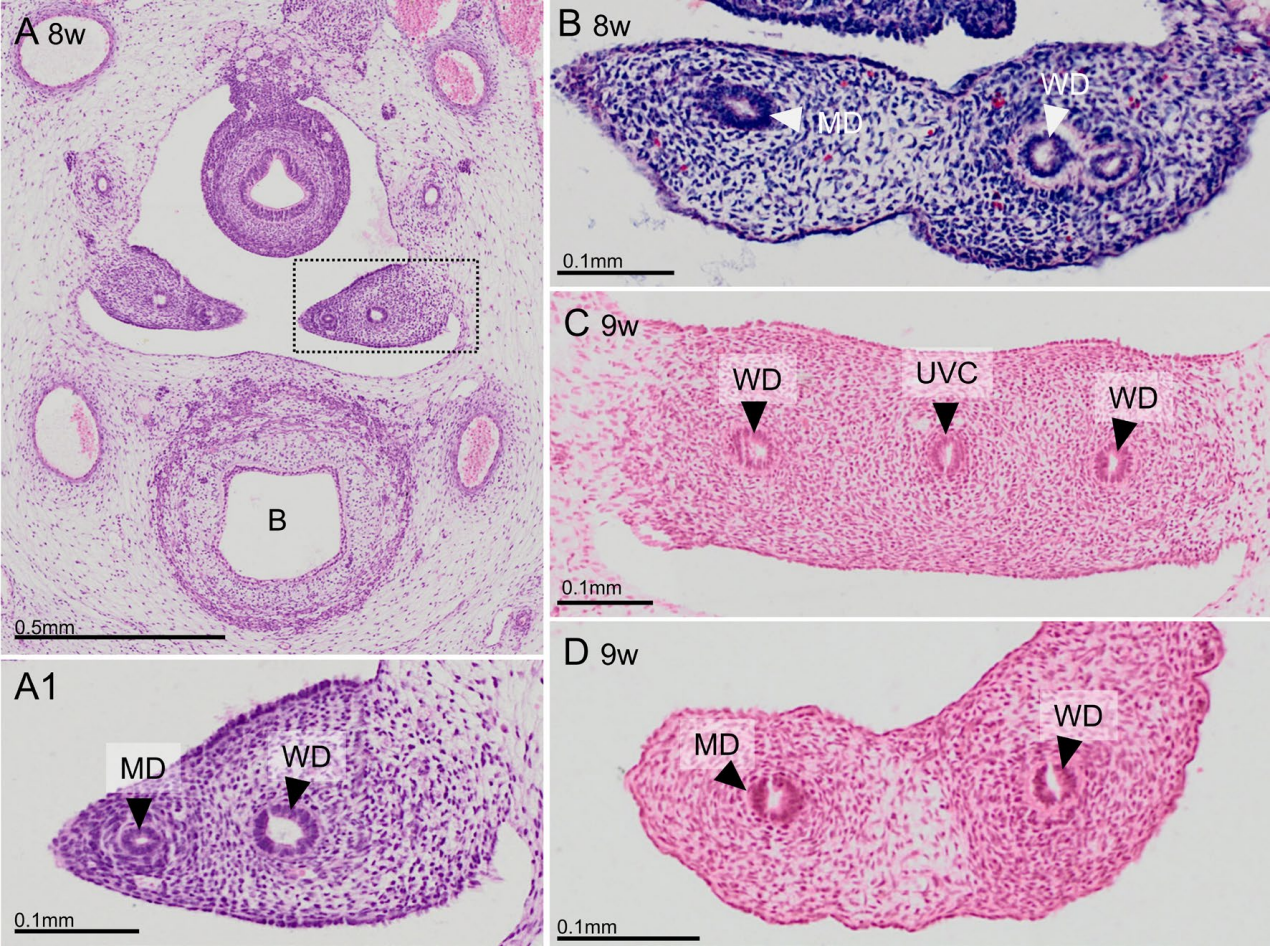
The mesenchymes surrounding the Müllerian and Wolffian ducts differ in architecture. The sections were taken just upstream (panels A (S48), B (S4141), and D (S89)) or downstream (panel C, S89) of the transition of the uterine tubes into the uterovaginal canal. The peri‐Müllerian mesenchyme differs from the peri‐Wolffian mesenchyme in having a more concentrically arranged, but less dense cell distribution (panels B and D). This mesenchymal arrangement is lost when the uterovaginal canal has formed. B: bladder; MD: Müllerian duct; UVC: uterovaginal canal; WD: Wolffian duct. Bars: 0.5 mm in panel A and 0.1 mm in all other panels.

The mesonephros and the mesonephric (“Wolffian”) duct begin to form at Carnegie stage (CS) 12 (~4.5 weeks of development) at the level of the upper thoracic segments (Hikspoors et al. 2015; Kruepunga et al. 2018). The Wolffian duct reaches the ventral cloaca at CS13 (Kruepunga et al. 2018). The longitudinal intraperitoneal ridge that contains the mesonephros and Wolffian ducts is known as the “urogenital ridge.” In CS15 embryos (~5 weeks of development), the initial steps of the development of the paramesonephric (“Müllerian”) ducts take place in the upper abdomen, between the bifurcation of the trachea cranially and the developing mesonephros caudally, and between the caudal cardinal veins dorsally and the main bronchi ventrally. At this site, the peritoneal epithelium thickens to form a square, frontally oriented, placode‐like structure (sides 130–150 μm; Figure 1A–D). This structure is accompanied on its caudomedial side by a dense mesenchymal ridge that extends laterally into the forming pleuro‐peritoneal fold (Figure 1A,C). The placode‐like structure soon acquires a corrugated surface (Figure 1D). Within this jagged epithelial landscape, the cranial ends of the Müllerian duct invaginate into the underlying mesenchyme to form the tubal entrance in early CS18 embryos (~6 weeks; Figure 1E–G) and extend their apex caudolaterally towards the Wolffian ducts (Figure 2A). The caudal extension of the Müllerian ducts was calculated to proceed at ~0.25 mm per Carnegie stage (≈2 days). During the formation of the tubal ostium, connective tissue cells separate the Müllerian and the Wolffian ducts (Figures 1E1 and 2A1). During its caudal extension, however, the tip of the Müllerian duct forms and elongates in close apposition to the adjacent Wolffian duct, with both ducts sharing a common, well‐developed, and continuous basement membrane (Figure 2A1–4). This intimate connection is seen only at the forming and extending caudal tip of the Müllerian duct, with connective tissue cells moving in to separate both ducts soon after the Müllerian duct has locally formed. Upon this separation, the Müllerian duct largely loses its basement membrane, whereas it is retained around the Wolffian duct (Figure 3B–D).

### 3.3 The Early Development of the Uterovaginal Canal

**FIGURE 4:**
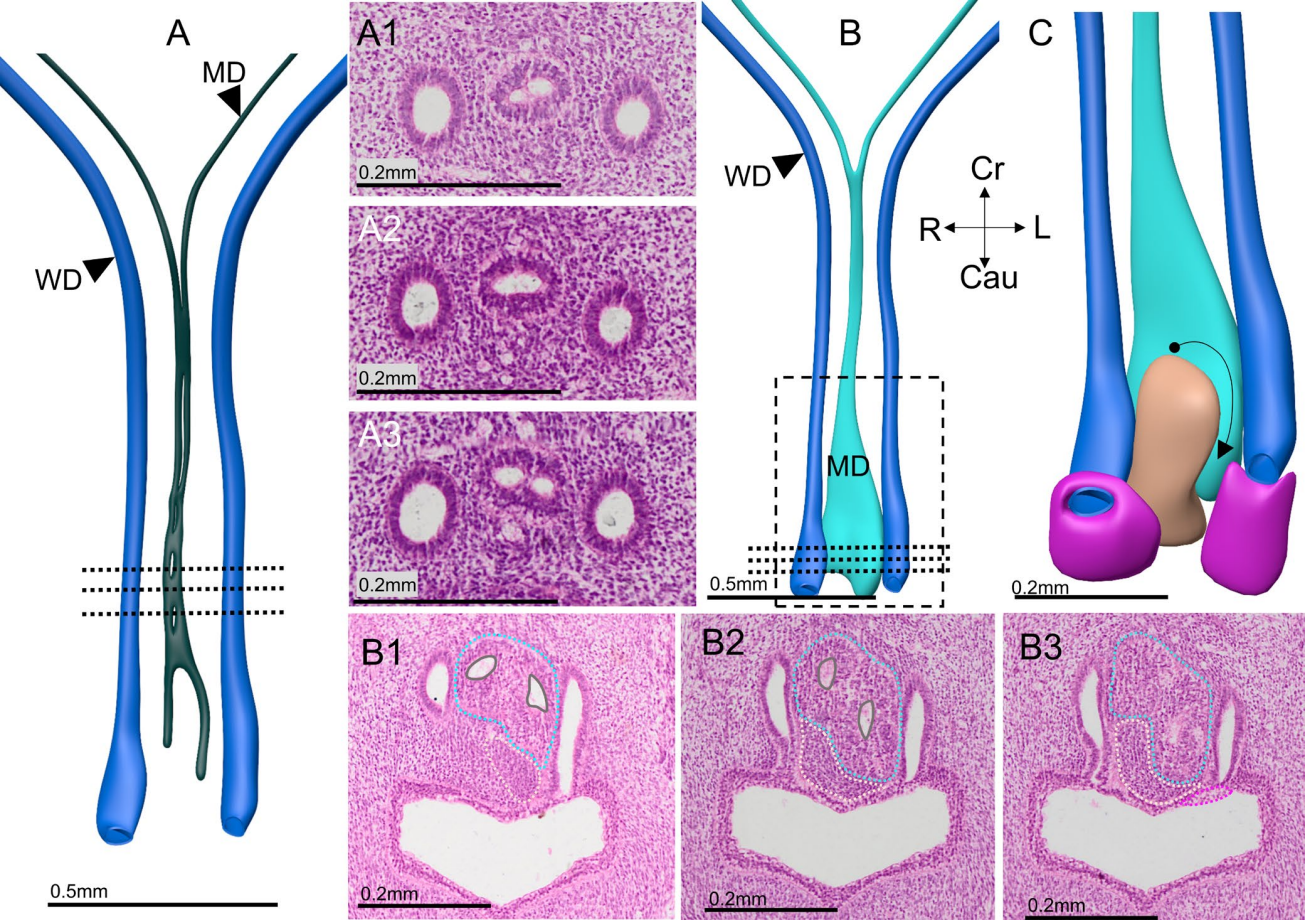
Shape and histology of the Müllerian and Wolffian ducts, and urogenital sinus at 8 weeks of development (embryo S48). Panel A shows the lumen of the Müllerian duct (dark gray) and the accompanying Wolffian ducts (dark blue). At the entrance to the lesser pelvis, the Müllerian ducts fuse to form the uterovaginal canal. All ducts are surrounded by a basement membrane, but the fusion of the uterovaginal canal is not yet complete (panels A1–3). Panel B shows the epithelial surface (cyan) of the caudal part of the same Müllerian duct shown in Panel A. At the caudal end, the Müllerian epithelium has thickened substantially (the “Müllerian head”; contours in panels B1–3), while the luminal diameter remains similar to that further cranial. A dense, mostly right‐sided mesenchymal wedge (pink contour) has formed asymmetrically between the Müllerian head and the urogenital sinus epithelium (pink contours in panels B1–3), while the left‐sided part of the Müllerian head pushes forward towards the urogenital sinus (panel C). Around the entry of the Wolffian ducts into the urogenital sinus, so‐called “clear cells” form the superficial layer of the epithelium of the urogenital sinus (pink cuffs in panel C and pink contours in panels B1–3). Cau: caudal; Cr: cranial; L: left; MD: Müllerian duct; R: right; WD: Wolffian duct. Bars: 0.5 mm in panels A and B, and 0.2 mm in all other panels.

The mesenchyme that envelops the Müllerian and Wolffian ducts differs markedly in cellular arrangement, with the Wolffian duct surrounded by the cellularly more compact mesenchyme (Figure 3B,D) and the Müllerian duct by more concentrically arranged cells (Figure 3A1,B,D). During Carnegie stage 22 (~7.5 weeks), when the Müllerian ducts have reached the caudal end of the mesonephros and approach the entrance to the lesser pelvis, both ducts and the urogenital ridge change course and extend medially to fuse with the corresponding structures of the opposite side at CS23 (~8 weeks of development; Figure 2B–E). The basement membrane on the facing sides of both approaching Müllerian ducts is even less developed than that covering the remaining part (Figure S2). Our observations in mouse embryos (next paragraph) indicate that the facing epithelial cells of both ducts intercalate and the structures themselves merge. Of the four human embryos of Carnegie stage 23 studied, three showed that both ducts had fused along a single continuous stretch, whereas one showed largely fused ducts with a few non‐fused stretches in between (Figure 4A). The multiple sites of incomplete fusion suggest that the Müllerian tubes form before they fuse (see also next paragraph). Due to the change in growth direction of the Müllerian ducts near their entrance to the lesser pelvis, the Wolffian ducts come to lie laterally to the Müllerian ducts (Figures 2E and 3). The frontally oriented septum that forms upon the fusion of the left and right urogenital ridges is known as the “broad ligament”, also known as the ligamentum latum (Figure 3C), while the fused part of the Müllerian ducts is known as the “uterovaginal canal”. The dense mesenchymal cuff that gradually comes to surround the uterovaginal canal and Wolffian ducts is first identifiable as a continuation of the peri‐Müllerian and peri‐Wolffian mesenchyme (Figure 3C,D) and becomes, as the “genital cord,” a prominent architectural feature of the uterovaginal canal. Caudally, the genital cord becomes less dense and ends near the Müllerian tubercle.

**FIGURE 5:**
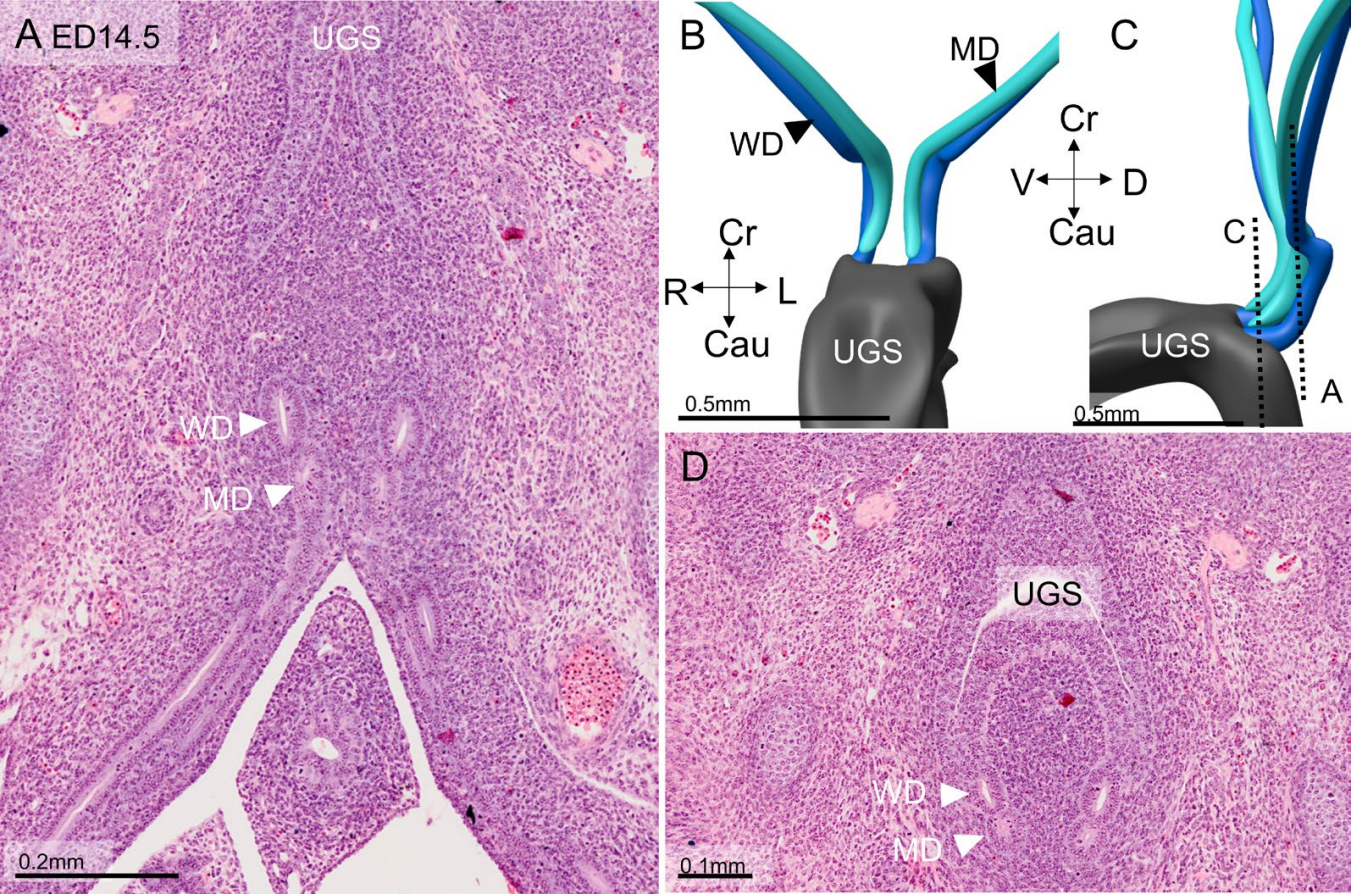
Shape and orientation of the Müllerian duct in an embryonic‐day 14.5 mouse embryo. Panels A and D show sections at the junction of the abdominal and pelvic parts of the Müllerian ducts (A) and near the Müllerian tubercle (D). Panels B and C show reconstructions of these areas. At this age, the Müllerian ducts have reached the urogenital sinus but must still fuse. Cau: caudal; Cr: cranial; D: dorsal; L: left; MD: Müllerian duct; R: right; UGS: urogenital sinus; V: ventral; WD: Wolffian duct. Bars: 0.2 mm in panel A, 0.1 mm in panel D, and 0.5 mm in the other panels.

**FIGURE 6:**
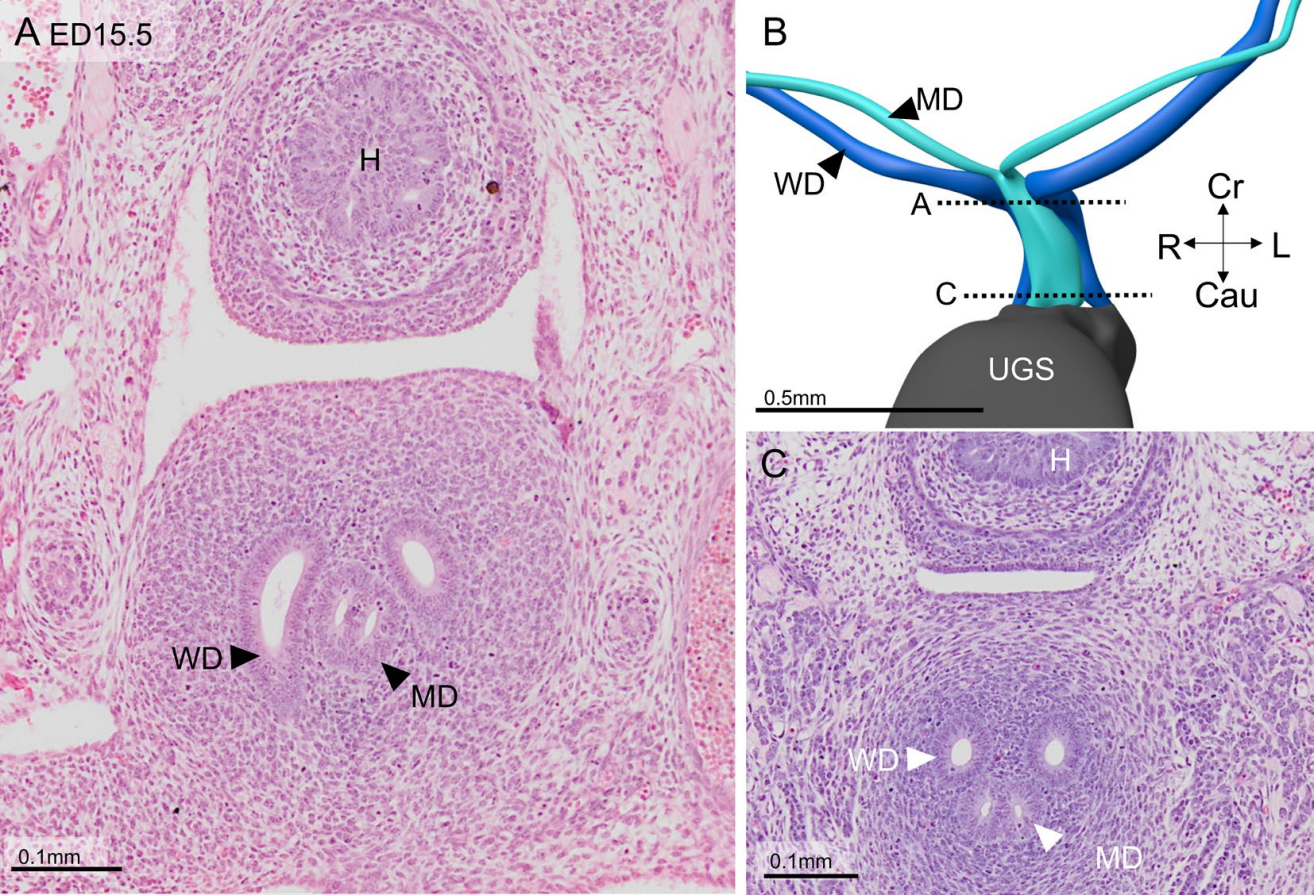
Shape and orientation of the Müllerian duct in an embryonic‐day 15.5 mouse embryo. Panels A and C show that the Müllerian ducts have fused and have a common basement membrane, but still have two lumina. Externally (panel B), a groove between both original ducts is still visible. Cau: caudal; Cr: cranial; H: hindgut; L: left; MD: Müllerian duct; R: right; WD: Wolffian duct. Bars: 0.1 mm in panels A and C, and 0.5 mm in panel B.

The morphology of the human female reproductive tract arguably differs from that of the mouse. Since the development of the uterine tubes is similar in both species (see the Discussion section), this difference mainly applies to the fate of the uterovaginal canal. We, therefore, also studied this structure in embryonic day 14.5 and 15.5 mice (comparable to ~7.5 and **~**8.5 weeks of human development). Figure 5 shows 2 sections and a reconstruction of the murine Müllerian and Wolffian ducts. In this mouse embryo, the intrapelvic parts of the Müllerian ducts have formed, but they have not yet fused to form the uterovaginal canal [for a comparable result, see Figure 2A in (Drews 2007)]. The reconstruction is almost indistinguishable from that shown of a human embryo in Figure 2E, except for the short delay between formation and fusion of the Müllerian ducts. One day later, both intrapelvic parts of the Müllerian duct have fused, but the adjacent walls of both ducts are still present as a merged epithelial septum along the entire length of the uterovaginal canal (Figure 6). The configuration of the Müllerian and Wolffian ducts at this stage is comparable to that shown in Figure 4. One more day later, a single uterovaginal canal has formed, except caudally, where, as in human embryos, 2 ducts remain (not shown). These data show that early Müllerian duct development, apart from the slightly delayed fusion of the intrapelvic Müllerian ducts, is largely comparable in human and mouse embryos.

### 3.4 The Early Fetal Development of the Uterovaginal Canal

Although an external groove in the epithelium indicates that the most caudal part of the Müllerian ducts does not fuse completely, only the local presence of two lumens demonstrates that fusion of the Müllerian ducts does not extend into its most caudal, sub‐sinusal end (Figures 2E1 and 4). This caudal bifurcation of the Müllerian ducts remains identifiable until ~10 weeks of development [described in the accompanying report (Hülsman, Köhler, et al. 2025c)]. The diameter of the uterovaginal canal is small (smaller than the accompanying Wolffian ducts) until it approaches the caudal bifurcation of its lumen (compare Figure 4, panels A1–3 and B1–3). Here, the wall of the Müllerian ducts gradually increases in thickness to form what we will describe as the “head” of the uterovaginal canal (*cf*. lumen in Figure 4A and external shape in Figure 4B). The epithelium of the caudal head is twice as thick as the cranial end of the uterovaginal canal and accounts for 30%–40% of its total length. Its caudal end touches the epithelium of the urogenital sinus ventrally and the Wolffian ducts laterally. The Wolffian ducts increase ~3‐fold in diameter when they approach the urogenital sinus. The contact between the widened caudal Müllerian and Wolffian epithelia is direct, with only a well‐developed basement membrane separating both structures (Figure 4B1–3). Ventrally, the bulging head of the Müllerian ducts elicits a shallow mount on the epithelium of the urogenital sinus [Figure 4B1–3; the “Müllerian tubercle” (O'Rahilly 1977)] or “Müllersche Wülste” (Vilas 1932). This conical elevation reaches its maximal diameter at ~9 weeks. The Müllerian tubercle locates at the transition of the relatively narrow upper (“pelvic”) urogenital sinus, which will form the vesical trigone and urethra, and the dorsoventrally larger lower (“phallic”) urogenital sinus, which will form the (vaginal) vestibule (Hülsman, Gao, et al. 2025b).

### 3.5 The Development of the Uterus

The mesenchymal condensation which surrounds the non‐fusing cranial part of the Müllerian and that which surrounds the corresponding Wolffian ducts develops as separate entities during the 8th and 9th weeks (Figure 3A1,B,D). This difference is barely visible in the genital cord, which is the dense mesenchyme that surrounds the fused, uterovaginal part of the Müllerian ducts (Figure 3C). During the 10th week of development, the smooth‐muscle layer becomes visible around the epithelium of the uterus and the uterine tubes (Figure S3).

**FIGURE 7:**
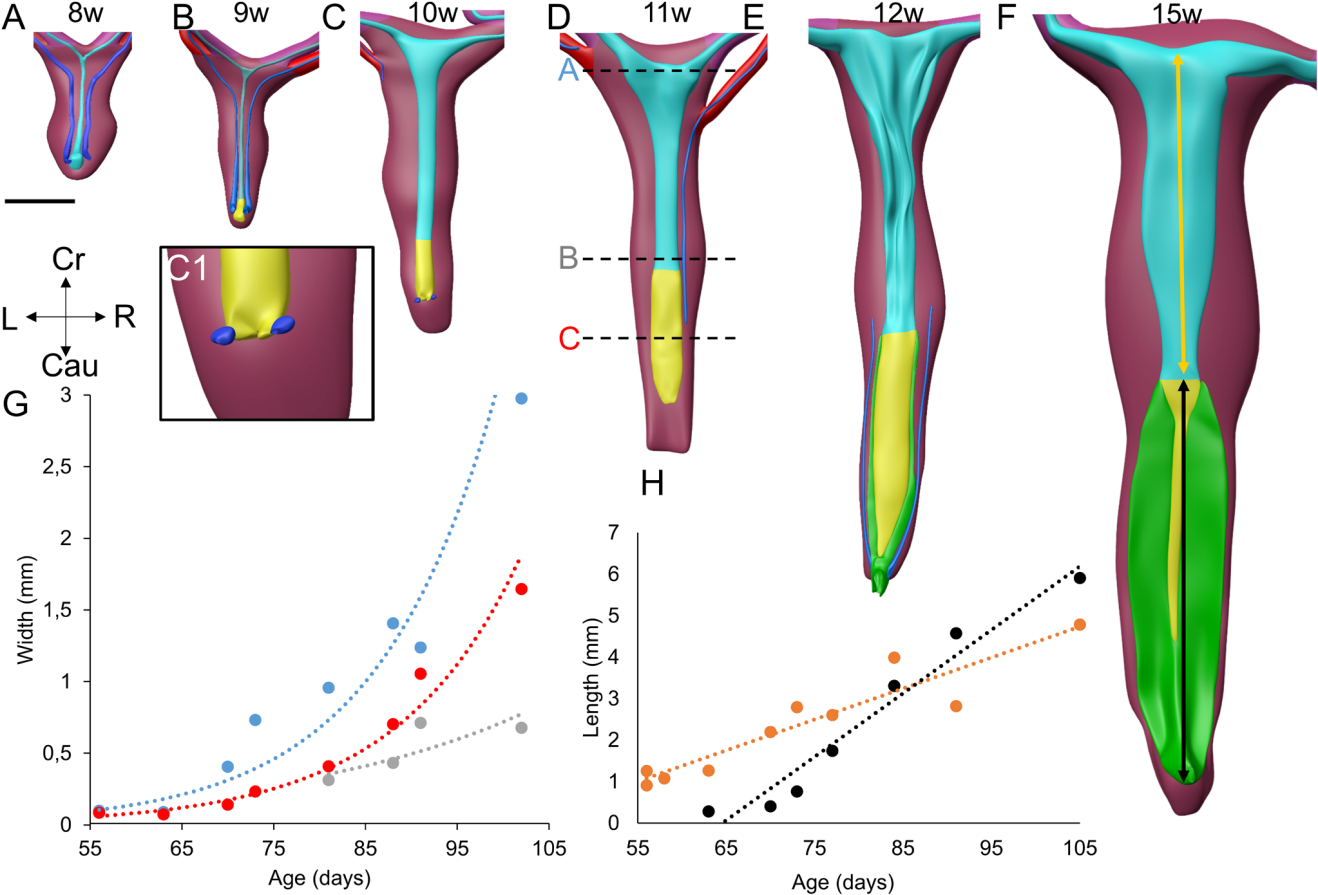
Growth and patterning of the uterovaginal canal. Panels A–F show the uterovaginal canal (cyan/yellow), uterine tubes (cyan), and genital cord (burgundy) between 8 and 15 weeks of development at the same magnification (S4141, S89, S1744, S1743, S2383 and S2392). Note that the uterovaginal canal grows both in length and width. The thin dark blue lines lateral to the Müllerian ducts are the regressing Wolffian ducts (see Figure 8 for images with more contrast). In the 10‐week‐old fetus, a short remnant of the Wolffian ducts is still present near its outlet into the urogenital sinus (see magnification, C1). The relatively thick mesenchymal collar in the middle part of the genital cord corresponds with the location of the developing uterine cervix (O'Rahilly 1977), while the caudal end of the collar would correspond with the uterovaginal junction. The width of the uterovaginal canal was measured at three different levels (“A,” “B,” and “C” in panel D). Width B could only be measured from 11 weeks) onwards. Panel G shows the increase of width with age. This graph demonstrates that the diameter of the lumen of the Müllerian ducts at positions A and C increases equally rapid, but drops ~2‐fold at position “B”. Panel H shows that the length of the vagina (black) increases ~2‐fold faster than that of the uterus (orange). Bars: 1 mm.

**FIGURE 8:**
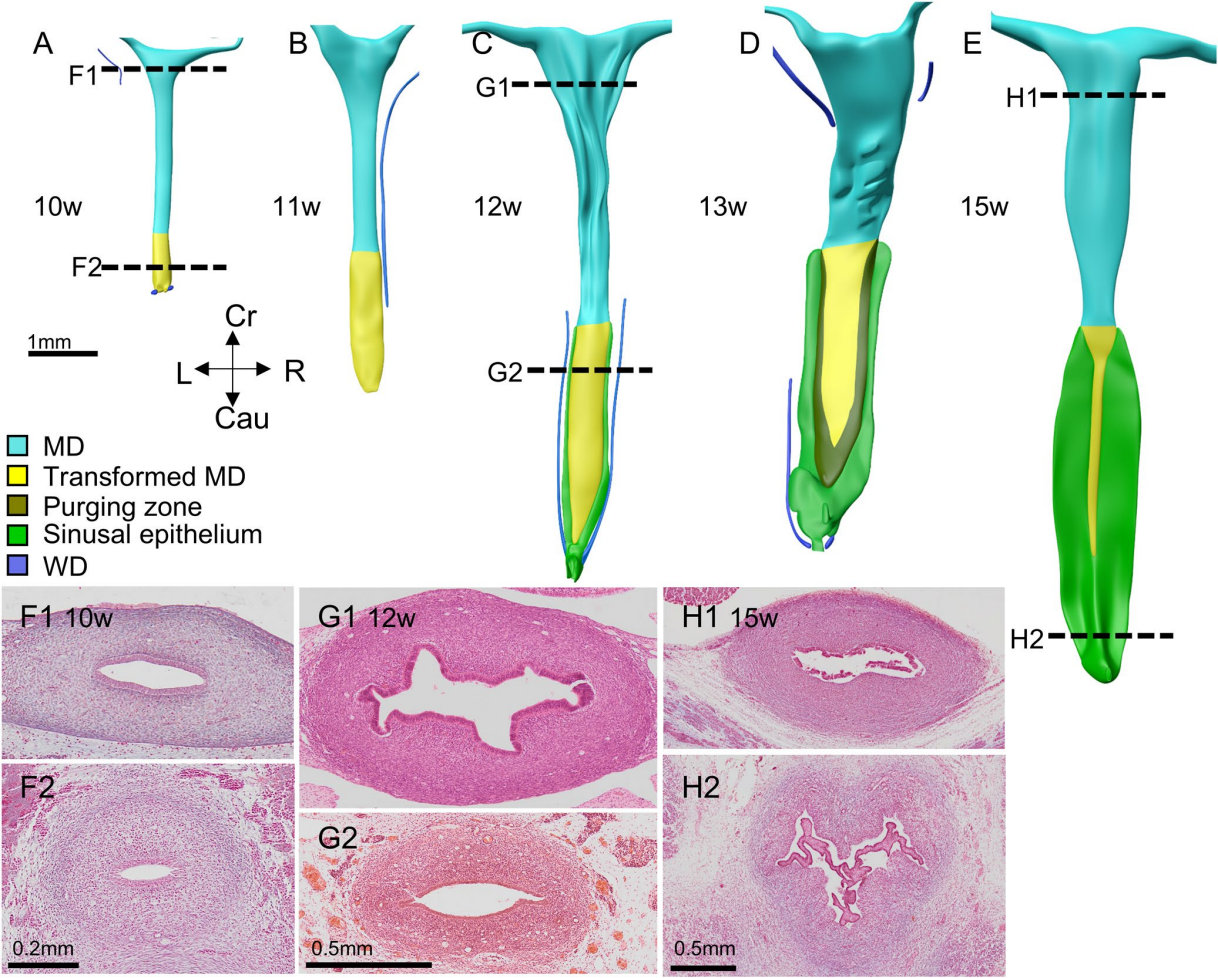
The luminal shape and histology of uterus and cervix between 10 and 15 weeks (S1744, S1743, S2383, S2212, and S2392). The fetal uterovaginal canals (panels A‐E) have the same color‐code as shown in Figure 7. Both shades of green show the vaginal plate and are described in the accompanying study (Hülsman, Köhler, et al. 2025c). In the stages shown (the location is indicated by dashed line in panels A, C, and E), the cervical canal is narrower and has a simpler shape (oval or wavy; panels F2 and G2) than the uterus, which develops longitudinal folds in its lumen (panels G1 and H1). Note that in the 15‐weeks fetus maceration detached the epithelium from the wall. Images of undamaged fetuses of this age class are available in literature (Meyer 1936) and confirm that the epithelium is attached at the mesenchymal wall of the uterovaginal canal. Cau: caudal; Cr: cranial; L: left; MD: Müllerian duct; R: Right: WD: Wolffian duct. Bars: 1 mm.

**FIGURE 9:**
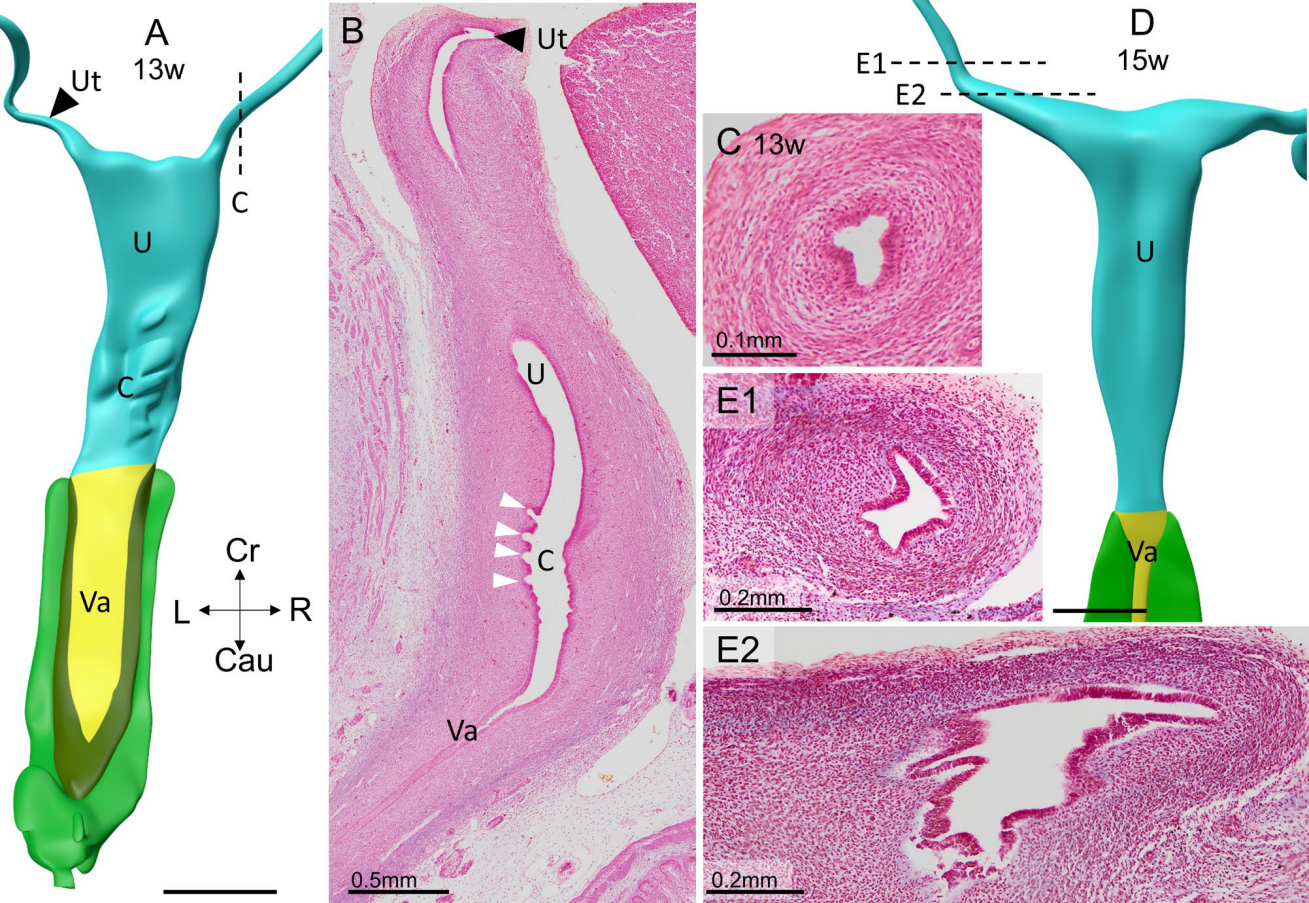
The palmate folds and the anteflexion of the uterus define the cervix at 13 weeks (fetus S2212). The lateral view of the reconstruction shows the normal bend between vagina and cervix (anteversion) and that between the cervix and uterus (anteflexion). The cervix has the same size as the uterus at this stage. Its palmate folds are visible as ridges of a washboard (arrowheads). The development of the vaginal epithelium (yellow and green in panel A) will be dealt with in the accompanying study (Hülsman, Köhler, et al. 2025c). The epithelium of the uterine tubes has folded and increased in diameter (panels C, E1, and E2) at 15 weeks of development (S2392). C: cervix; Cau: caudal; Cr: cranial; L: left; R: right; U: uterus; Va: Vagina. Bars: 1 mm.

The cranial part of the epithelium of the uterovaginal canal is uniform Müllerian epithelium up to Carnegie stage 23 (8 weeks; cyan part of tube in Figure 7A), and is replaced in the vagina by urogenital epithelium (yellow) during the next 8 weeks (Figure 7A‐F; reported in detail in our accompanying study (Hülsman, Köhler, et al. 2025c)). In this period, the vagina (yellow) grows ~2‐fold faster than the uterus (cyan) (Figure 7H). The waist that forms between the cranial and middle parts of the genital cord (Figure 7A–G) corresponds roughly with the junction between the body and the cervix of the uterus (Koff 1933; O'Rahilly 1977). The wall of the uterus at 12 weeks has formed coarse folds (Figure 8C,G1). Unfortunately, the condition of the epithelium was not good enough to assess this feature in the 15‐weeks‐old specimen. The development of a ventrally open bend at the junction between the body and cervix of the uterus, which is known as the uterine anteflexion, becomes identifiable at ~13 weeks of development (Figure 9B). The clinically relevant intra‐ or extraperitoneal topography of the uterus is complex. The cranial 15%–20% of the uterovaginal canal lies intraperitoneally during the entire period studied (8–15 weeks). Assuming that the vaginal component of the uterovaginal canal accounts for 45%–50% of its total length (Koff 1933), the uterus, but not the cervix, is largely covered by peritoneal epithelium.

### 3.6 The Development and Topography of the Gubernaculum

**FIGURE 10:**
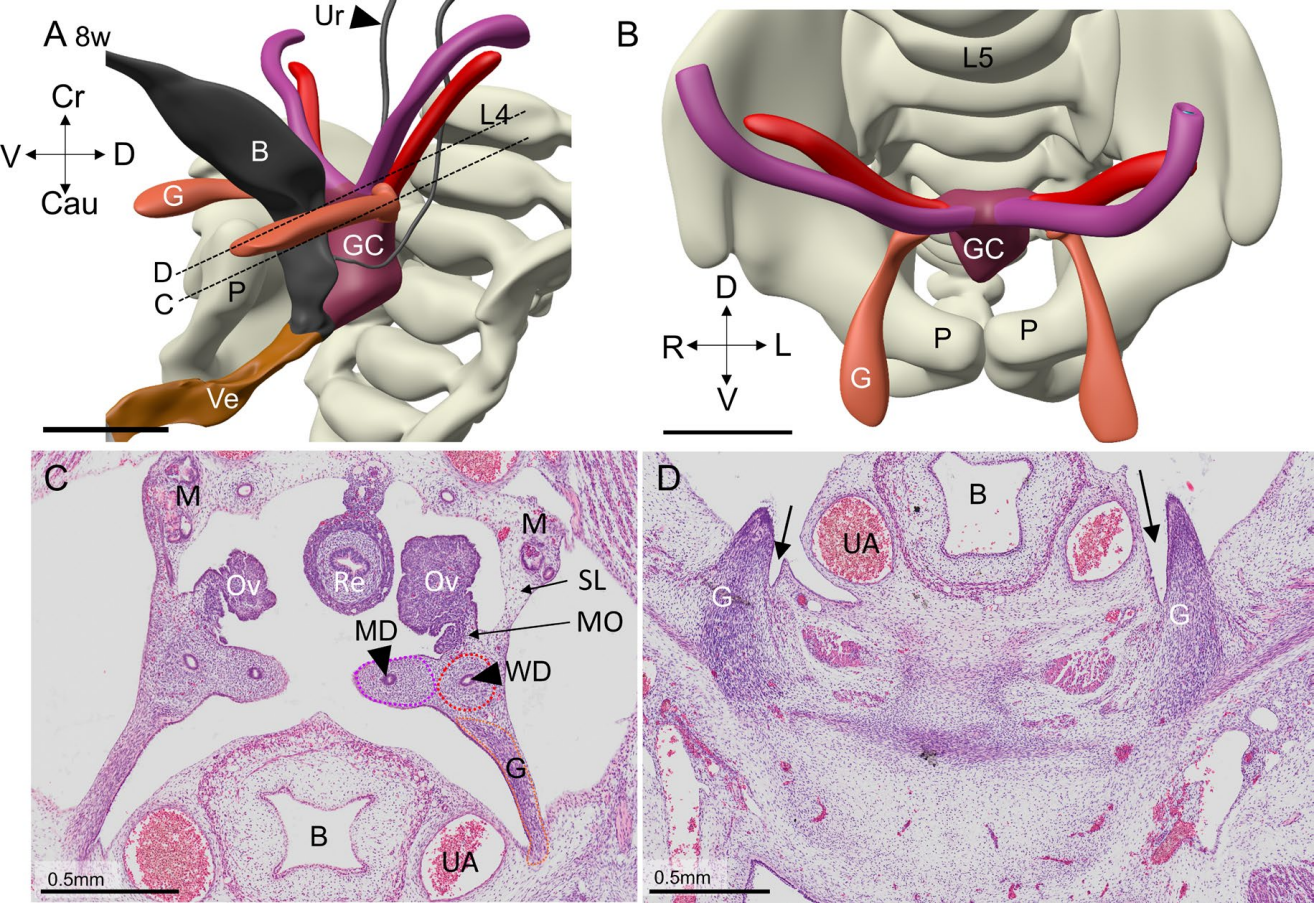
The histology and topography of the gubernaculum and the proper ovarian ligament at the end of the indifferent period at 8 weeks of development (embryo S4141). Panel A shows a ventral‐left view of the urogenital system and the vertebral column, and panel B a cranial view of the same structures. Panels C and D show sections of the gubernaculum where it is attached to the mesenchyme surrounding the Wolffian duct (panel C) and where it fans out widely in the ventral body wall, just medial to the oblique abdominal muscles (panel D). The gubernaculum is characterized at this stage by the abundant presence of smooth muscle cells (panel D). The histological make‐up of the suspensory ligament of the ovary is dominated by the presence of the persisting parts of the caudal mesonephros. Panel C clearly shows that the peri‐Wolffian mesenchyme intervenes between the gubernaculum ventrally and the suspensory ligament and mesovary dorsally. B: bladder; Cau: caudal; Cr: cranial; D: dorsal; G: gubernaculum; GC: genital cord; L: left; MD: Müllerian duct; MO: mesovary; P: pubic bone; R: right; SL: suspensory ligament of ovary; UA: umbilical artery; Ur: ureter; V: ventral. Bars: 1 mm.

In 6.5‐week‐old embryos (Carnegie stage 18–19), the gubernaculum is first visible as a dense mesenchymal structure in the tissue bridge that connects the caudal part of the urogenital ridge and the ventrolateral body wall. The gubernaculum is found in the lateral part of the tissue bridge (Figure S4). At 7 and 8 weeks of development, it medially attaches to the mesenchymal sleeve that surrounds the Wolffian duct (Figures 10C, S4 and S5) just cranial of the cranial beginning of the uterovaginal canal (Figure 10A). For this reason, it is a convenient landmark to separate the uterine tube from the uterus (van der Schoot, 1996). Ventrolaterally, the gubernaculum fans out in the body wall (Figure 10B,D, and Figures 4 and 5). The subsequent development of the gubernaculum reflects its evolving attachment to the lateral body wall (Mekonen et al. 2015): it expands in a caudolateral and then in a ventromedial direction. In the 8‐week embryo, the lateral and ventral parts of the gubernaculum are surrounded by an initially shallow excavation of the peritoneal cavity, the forming processus vaginalis or canal of Nuck (arrows in Figures 10D, S5, and S6).

**FIGURE 11:**
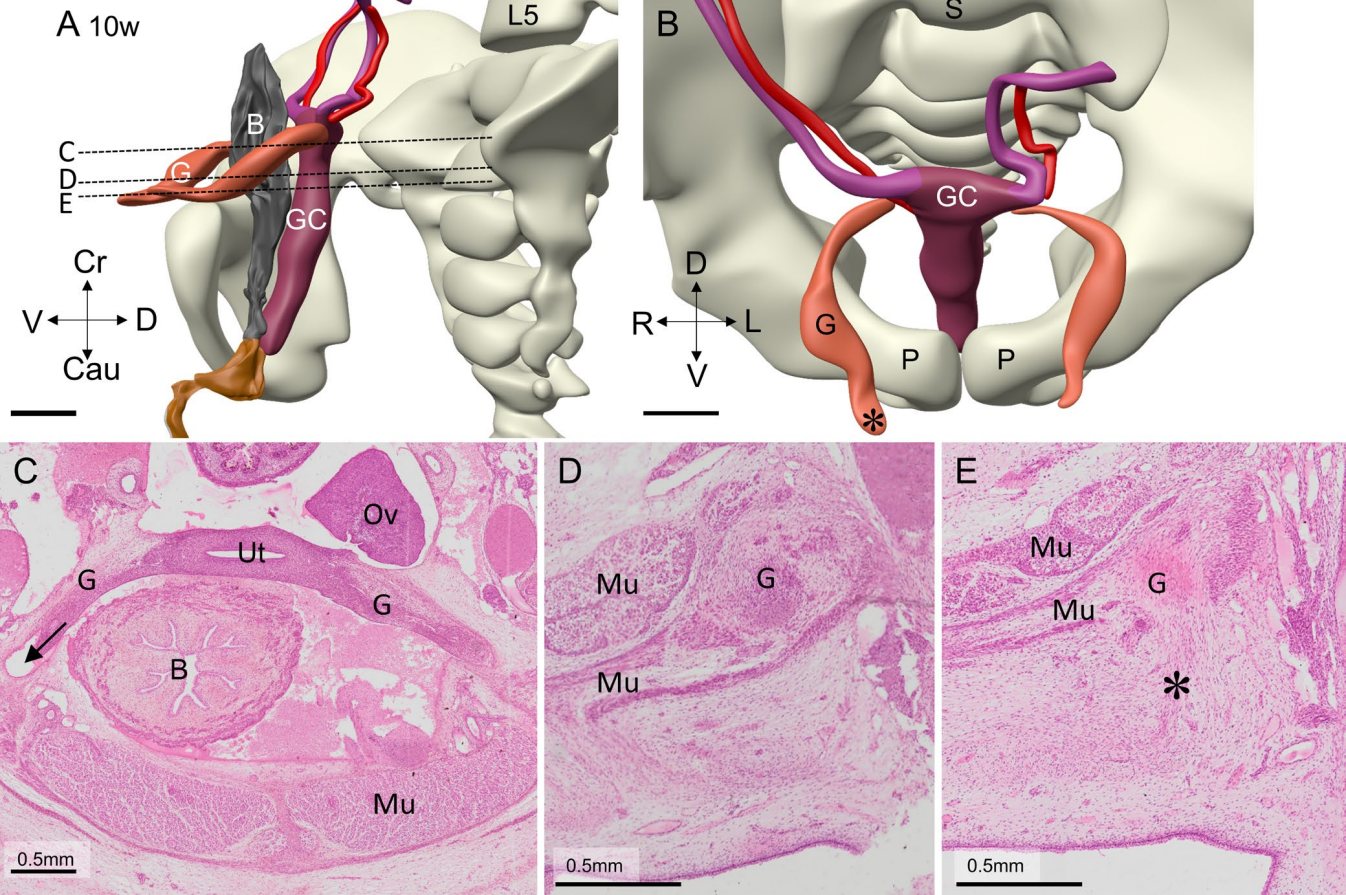
The architecture and topography of the gubernaculum, vaginal process, and suspensory ligament at 10 weeks (fetus S1744). Panel A shows a ventral‐left view of the urogenital system and the vertebral column and panel B a cranial view of the same structures. Panels C–E are histological sections at the level indicated in panel A. The gubernaculum can be found within the vaginal process (canal of Nuck) in panel C (arrow), passes into the retroperitoneum in panel D, and enters the inguinal canal in panel E. Note the muscles of the abdominal wall (Mu). Asterisk: subcutaneous part of gubernaculum. B: bladder; Cau: caudal; Cr: cranial; D: dorsal; G: gubernaculum; GC: genital cord; L: left; MD: Müllerian duct; P: pubic bone; R: Right; S: sacrum; V: ventral. Bars: 1 mm.

**FIGURE 12:**
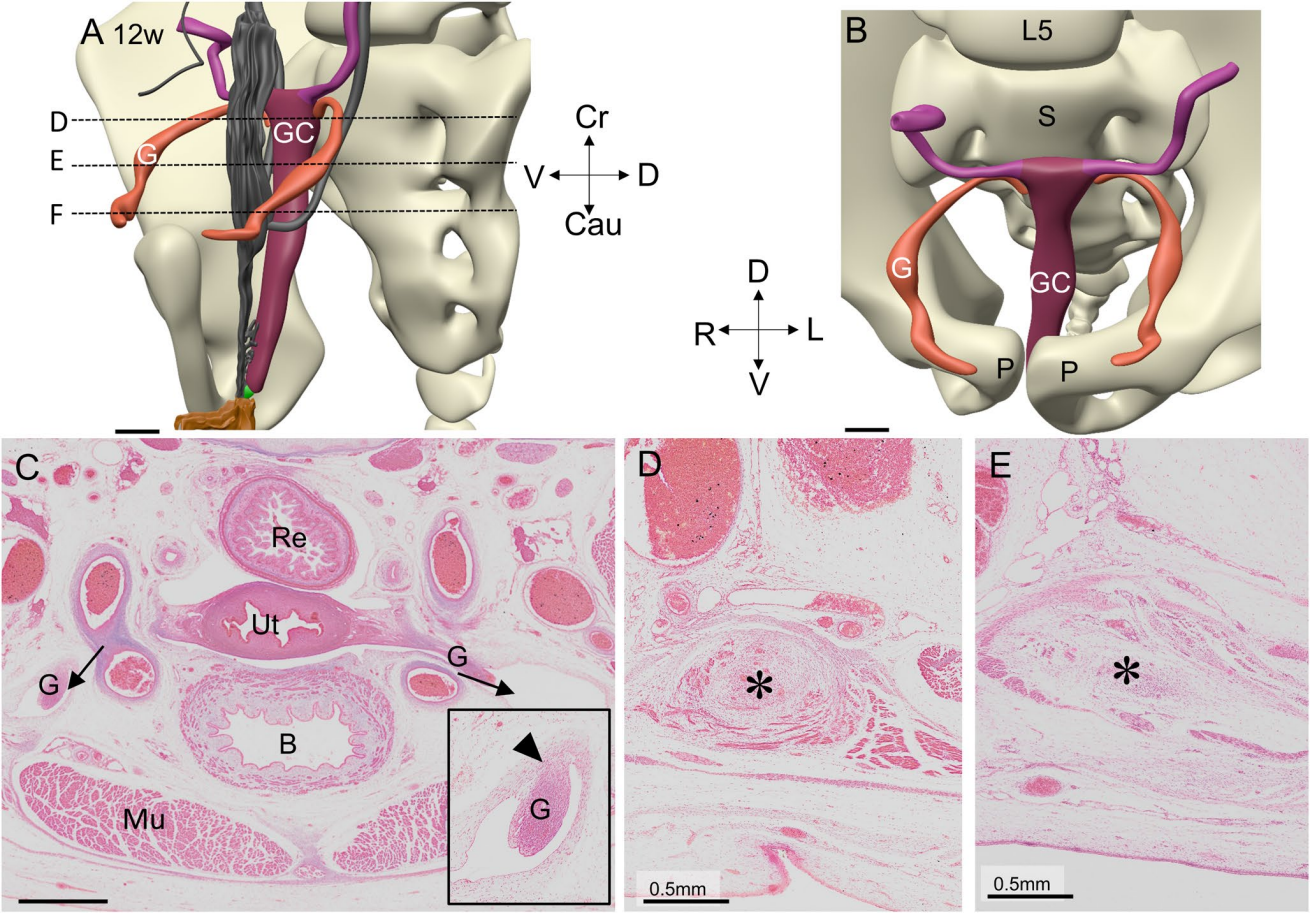
The architecture and topography of the gubernaculum, vaginal process, and suspensory ligament at 12 weeks (fetus S2383). Panels A and B are left ventral views of the genital cord with the gubernaculum. Panel A has some reference structures included, such as the vertebral column and urogenital system. Panel C is a cranial view of the genital cord and gubernaculum with the pelvic bones and vertebral column. In panel D, the gubernaculum can be found within the canal of Nuck (arrows), in which the gubernaculum has a dorsal mesentery (arrowhead in magnification) (Panels E and F). The canal of Nuck courses inferiorly along the ventrolateral body wall and ends in the subcutaneous tissue of the mons pubis. Asterisks in panels E and F: extraperitoneal part of gubernaculum. B: bladder; Cau: caudal; Cr: cranial; D: dorsal; G: gubernaculum; GC: genital cord; L: left; MD: Müllerian duct; P: Pubic bone; Mu: abdominal muscles; R: right; Re: rectum; S: sacrum; Ut: uterus; V: ventral. Bars: 1 mm.

Between 8 and 12 weeks, gubernacular differentiation proceeds intraperitoneally inside the ventrolateral tubular evagination of the peritoneal cavity, the canal of Nuck (arrows in Figures 10D, 11C, 12D, S6, and S7A). Inside the canal of Nuck, the gubernaculum is dorsally attached to the adjacent tissue by a mesentery‐like structure (inserts in Figure 12D and Figure S7A). The canal of Nuck is initially widest in its cranial part (Figure S8C,D), but gradually narrows in that area, while the gubernaculum assumes the phenotypic properties of the round ligament (Figures 11C–E and 12D–F; Figures S7B and S8C,D). The canal of Nuck ends caudally between the straight and oblique internal abdominal muscles, that is, near the abdominal entrance to the future internal inguinal canal. The terminal fibers of the gubernaculum are, therefore, extraperitoneal structures that pass ventrally between the straight and internal oblique abdominal muscles and through the aponeurosis of the external oblique muscle into the subcutaneous tissue of the mons pubis (Figures 10D, 11D,E, and 12E,F, and Figures S6, S7B and S8D). The transition of the intraperitoneal to the retroperitoneal course is seen in Figure 12E,F, and Figure S8C. More caudally, the interstitial part of the round ligament breaches the tendinous fascia of the external oblique muscle and spreads in the subcutaneous space of the mons pubis (Figures 10C,D and 11D,E, and Figures [Link], [Link], and S8D). Here, the distribution of the components of the round ligament becomes increasingly mottled. The attachment of the smooth muscle strands of the gubernaculum to the ventral body wall is fan‐shaped, with pronounced widening and mottling of its histological details towards its caudoventral end. Its caudomedial end is, therefore, difficult to locate [asterisks in Figures 11E, 12F, and S7B, (Attah and Hutson 1991)]. The body of the uterus remains encased by the broad ligament. Near its cranial end, the broad ligament continues lateroventrally into the gubernaculum (Figure 11C) and laterodorsally into the suspensory ligament of the ovary and the mesovarium.

The Wolffian ducts and their surrounding mesenchyme are also attached dorsolaterally to the dorsal body wall. This attachment, the suspensory ligament of the ovary, consists mostly of loose mesenchyme and contains the caudal remnants of the mesonephros (glomeruli and ducts; Figure 10C). The mesovary, the mesentery of the ovary, is located on the dorsal side of the broad ligament. The proper ovarian ligament, which should be located between the ovary and the tubouterine junction, appears to be still a non‐discernible portion of the mesovary. These data show that, although the adult proper ovarian ligament appears to be a continuation of the gubernaculum (Attah and Hutson 1991), it attaches opposite the gubernaculum to the peri‐Wolffian mesenchyme and is not histologically continuous with the mostly smooth‐muscular gubernaculum (Figures 10C and S4C).

### 3.7 The Uterine Tubes

**FIGURE 13:**
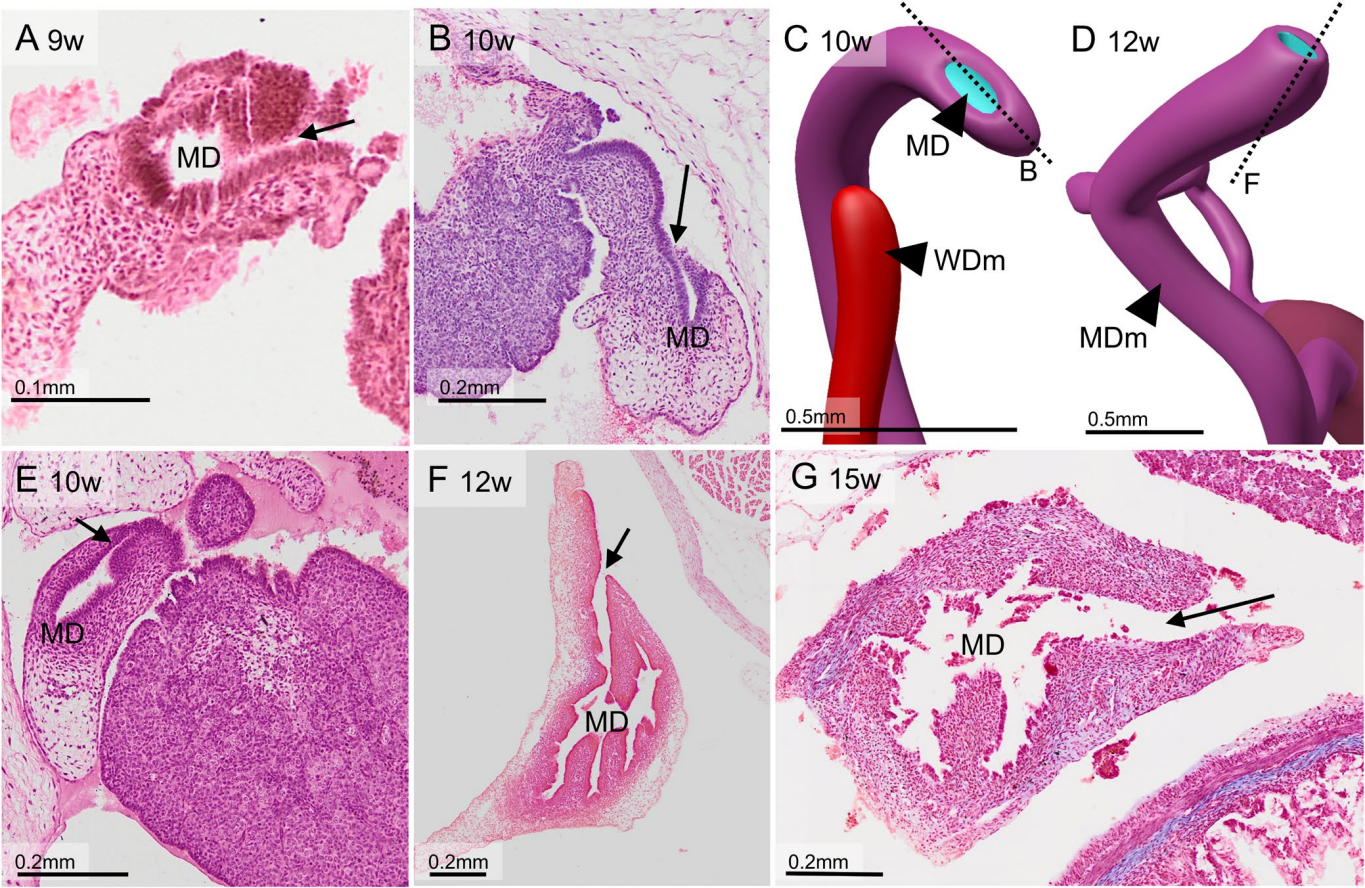
Development of the abdominal ostium of the Müllerian duct between 9 and 15 weeks of development. Panels C and D show reconstructions of the Müllerian ostium at 10 and 12 weeks of development (S4908 and S2383). The ostium consists of the Müllerian‐duct epithelium (cyan) and its mesenchymal cuff (purple). The Wolffian duct has its own mesenchymal cuff (red). Panels A, B, and E–G are sections of the Müllerian ostium at the indicated developmental age. The plane of sectioning in B and F are indicated in panels C and D, respectively. Until 10 weeks the tubal lumen and ostium are smooth (panels A, B, and E). Thereafter, the lumen of the duct becomes more folded (panels F and G). Maceration has damaged the epithelium of the ~15 weeks specimen (panel G) MD: Müllerian duct; MDm: Müllerian duct mesenchyme; WDm: Wolffian duct mesenchyme. Bars: 0.1 mm in panel A, 0.5 mm in panels C and D, and 0.2 mm in the other panels.

Although the ostium of the Müllerian duct at 6 weeks of development has seemingly formed a fimbria‐like structure of its abdominal ostium (Figure 1F,G), fimbriae remain subsequently unidentifiable until at least 15 weeks of development (Figure 13). Between 10–12 weeks, the lumen of the uterine tubes forms many epithelial folds (Figures 9C,E and 13; Figure S9). The uterine tube is embedded in a core of dense mesenchyme and a superficial sleeve of loose mesenchymal tissue. This sleeve continues into the suspensory ligament of the ovary (Figure S4B,C), which still harbors remnants of the mesonephros at its root (Figure 10C). At ~12–15 weeks of development, a smooth muscle layer forms in the dense mesenchymal sleeve around the uterine tubes (Figures 9C,E1,E2 and S9).

### 3.8 The Topographic Position of Uterus and Vagina in the Pelvis

**FIGURE 14:**
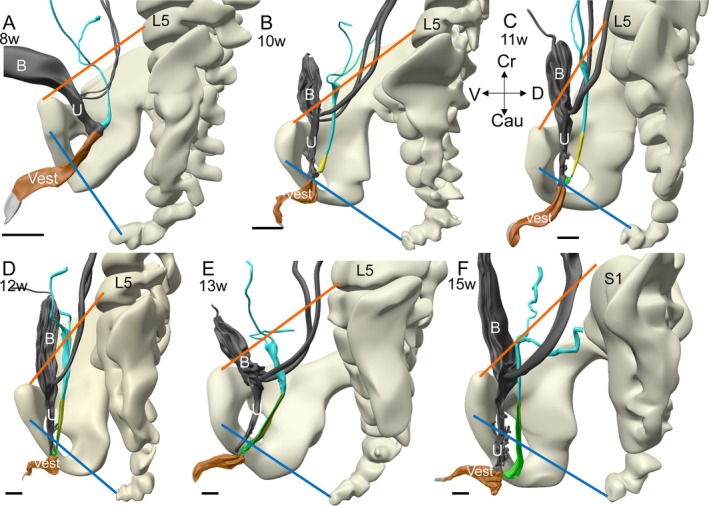
Growth and position of the Müllerian duct relative to the vertebral column and symphysis. The panels show left‐lateral views of the pelvic region including vertebrae, right pelvic bone, Müllerian duct (cyan), bladder and urethra (gray) and vaginal vestibule (brown). The orange line represents the pubolumbar line, which connects the cranial border of the symphysis with the middle of the body of lumbar vertebra 5 (L5). At all stages investigated, this line touches the cranial border of the uterovaginal canal. Note that in the 15 weeks fetus, L5 is not present, so that the pubolumbar line was made to pass above sacral vertebra 1 (S1). The blue line represents the pubococcygeal line, which reflects the position of the pelvic floor (Betschart et al. 2013). The caudal end of the Müllerian duct extends and passes this line at 12 weeks of development, which positions the Müllerian orifice in the subcutaneous perineal space. The Figure further shows that the craniocaudal growth of the urethra and the Müllerian duct are similar. B: bladder; Cau: caudal; Cr: cranial; D: dorsal; L5: lumbar vertebra 5; S1: sacral vertebra 1; U: urethra; V: ventral; Vest: vaginal vestibule. Bars: 1 mm.

The junction between the uterovaginal canal and the developing uterine tubes remains located on the pubolumbar line from the 8th to at least the 15th week of development (orange line, Figure 14). This line, which connects the cranial border of the symphysis with the 5th lumbar vertebra, still touches the most cranial point of the uterus in adult specimens, showing that it can be used as a developmental landmark. The pubococcygeal line represents the position of the pelvic floor in the adult (Betschart et al. 2013). Caudally, the Müllerian tubercle lies just cranial to the pubococcygeal line up to the 11th week of development (blue line in Figure 14). After 11 weeks of development, the urethra and vagina grow faster in the craniocaudal direction than the surrounding pelvic bones, so that at 15 weeks, the caudal 30%–35% of the pelvic organs lie caudal to the caudal boundary of the lesser pelvis [Figure 14F; (Hülsman, Gao, et al. 2025a)]. In the 7 weeks between the onset of Müllerian‐duct fusion (CS22; ~7.5 weeks) and its arrival at the definitive position (~15 weeks), the length of the common part of both Müllerian ducts has increased ~10‐fold (orange line, Figure 2F).

## 4 Discussion

Our findings show that the development of the cranial portion of the female genital tract (uterine tubes and uterus) can be best divided into intraabdominal and intrapelvic parts. The intrapelvic part comprises both the uterus and the vagina. We describe the development of the vagina, in particular the replacement of its Müllerian by urogenital epithelium, separately in the accompanying report (Hülsman, Köhler, et al. 2025c). Our observations on early Müllerian duct development in the abdominal cavity reveal an extensive similarity between humans and animal models, in particular the mouse. The appraisal of the development of the uterovaginal canal in mice and humans unexpectedly showed that early development of the intrapelvic part of the Müllerian ducts in mouse embryos is largely comparable to that in human embryos, and that the perceived differences between both species may well be traced to regional growth differences of the fundic part of the uterus near the tubouterine junction.

### 4.1 Growth of the Intraabdominal Müllerian Ducts

The development of the Müllerian ducts proceeds from specification and “placode‐like” structure during Carnegie stages 15–16, via epithelial invagination and funnel formation during stages CS17‐18, to proliferation and elongation to reach the definitive topography in Carnegie stages 20–23 [Figures 1, 2, 4; (Orvis and Behringer 2007; Roly et al. 2018; Santana Gonzalez et al. 2021)]. The progenitor cell‐containing epithelial thickening forms at the level of the 3rd thoracic vertebra, just caudal of the limb bud (arm) at the cranial end of the mesonephros. Its close topographic relation to the pleuroperitoneal fold (Figure 1A–D) is maintained during subsequent embryonic development. The thick placode‐like cell plate undergoes partial epithelial‐to‐mesenchymal transition in the 6th week. This transition allows the cell plate to form an increasingly rugged surface and, subsequently, to invaginate into the underlying mesenchyme of the urogenital ridge and form the fimbriated infundibulum of the Müllerian duct [Figure 1G; (Yang et al. 2020; Santana Gonzalez et al. 2021)]. This early phase of Müllerian‐duct development, which ends with the Müllerian duct contacting the Wolffian duct is Wolffian‐duct independent (Carroll et al. 2005; Kobayashi et al. 2004).

The non‐luminal, wedge‐shaped apex of the invaginating Müllerian duct extends caudally between the Wolffian duct and the peritoneal epithelium of the urogenital ridge [Figure 2A1–4; (Gruenwald 1941; Burkl and Politzer 1953; Frutiger 1969)]. Unexpectedly, however, the solid epithelial tip of the Müllerian duct does not show cell proliferation, whereas nearby Wolffian epithelial cells do (Burkl and Politzer 1953; Ludwig 1998). It was hypothesized, therefore, that proliferating Wolffian cells would contribute to the Müllerian ducts because both ducts were encased in a common basement membrane, whereas the basement membrane between the adjacent parts of the Müllerian and Wolffian ducts had disappeared (Figure 2A). Transfer of Wolffian epithelial cells to the growing Müllerian duct could be excluded because Wolffian epithelial cells can be selectively stained for the presence of, for example, cytokeratin 8 and E‐cadherin, and can be selectively targeted by the Hoxb7 promoter in mice (Orvis and Behringer 2007). How the Müllerian ducts recruit cells to extend caudally must still be shown, therefore. Once a new piece of the Müllerian duct has formed, this piece acquires a lumen and separates away from the Wolffian duct because mesenchyme, partly originating in the coelomic epithelium (Orvis and Behringer 2007), is deposited in between (Fujino et al. 2009). The mesenchymes surrounding the Wolffian and Müllerian ducts differ with respect to their cell density and arrangement (the Wolffian duct is surrounded by denser mesenchyme), whereas mesenchymal cells in the Müllerian duct are more concentrically arranged [Figure 3A1,B,D; (Arango et al. 2008; Haraguchi et al. 2021; Zhao et al. 2022)], their gene expression profiles [for a review, see (Mullen and Behringer 2014)], and their hormonal responsiveness (Crocoll et al. 1998; Drews et al. 2001; Murashima et al. 2011). Due to the similarities in early uterine‐tube development between birds and mammals, and between mice and men (Gruenwald 1941; Jacob et al. 1999), there is a fair degree of consensus about the mechanisms of development of the uterine tubes. Accordingly, our morphological findings with respect to human uterine‐tube development are largely in agreement with earlier human studies (Burkl and Politzer 1953; Hashimoto 2003; Smith et al. 2010). Instructive images of the Müllerian placode‐like structure in human embryos are provided, in addition to our Figure 1, by Burkl and Politzer (1953) and Frutiger (1969).

### 4.2 The Intrapelvic Formation and Development of the Müllerian Ducts

Müllerian‐duct formation passes as a wave along the Wolffian duct. The wave breaks in the pelvic part of the Müllerian ducts. Here, these ducts form and fuse in the cranial direction (Koff 1933; Hashimoto 2003). Our observations in the mouse (see next paragraph) show that the ducts first form and only then fuse to form the uterovaginal canal during Carnegie stages 22–23 (Figure 2B–E). Fusion does not occur caudally. To permit their fusion in the midline, both Müllerian ducts change their course in the medial direction where they emerge from the caudal mesonephros. The topographic position of the most cranial part of the newly formed uterovaginal canal is very reliably located on the pubolumbar line between the cranial edge of the symphysis and the 5th lumbar vertebra (Figure 14, pelvic bones).

### 4.3 Early Intrapelvic Müllerian Duct Development Is Similar in Mice and Man

Although it is now well established that the most caudal part of the human Müllerian ducts does not fuse [Figure 3; (Kempermann 1931; Koff 1933; Hashimoto 2003], the formation of the more cranial part of the uterovaginal canal is described in much less detail. Our observations suggest that fusion into a single canal follows the formation of two separate Müllerian ducts in the lesser pelvis. The disappearance of the dorsoventrally oriented septum in the uterovaginal canal is the last step. In human embryos, ductal formation, fusion, and the forming of a single lumen succeed each other rapidly, but the steps seem temporally better resolved in mouse embryos. Although the female genital tract of rodents differs in part from that in humans, the formation of the initially still septated uterovaginal canal (Harada and Akita 2023) and its ligaments (Iwanaga et al. 2016) appears similar and instructive for the human situation: the left and right intrapelvic Müllerian ducts both form in embryonic day 14.5 mice (equivalent to Carnegie stage 21) and contact the Müllerian tubercle, but still have separate lumens along their entire course [Figure 5; fig. 2A in Drews 2007)]. The adjacent parts of both ducts fuse or merge during the next day (Carnegie stage 22) to form the murine uterovaginal canal, but the 2 lumens remain present for another day (equivalent to Carnegie stage 23; Figure 6; fig. 1K–P in Harada and Akita 2023). The sequential steps in the formation of the murine uterovaginal canal suggest that the remnants of the septum that we observed in a CS23 human embryo are the remains of a similar septum in the mouse (fig. 2 in Harada and Akita 2023). The conclusion that the formation of the uterovaginal canal of rodents is very similar to that in human embryos (Figures 5 and 6) indicates that the duplex uterus of rodents (with two long uterine horns) does not arise as a consequence of minimal fusion of the Müllerian ducts (Mullen and Behringer 2014), but as a consequence of differential growth of a small, non‐fused uterine portion near the tubo‐uterine junction. Evidence in favor of this hypothesis shows that the transverse “middle” segment of the Müllerian ducts, which connects the “proximal” (gonadal) and “caudal” (uterovaginal) segments, grows fastest in mice (Nakajima et al. 2019). This relatively short middle segment corresponds with the uterine fundus. Anomalies of the fundus (arcuate or (sub‐)septate uteri) are ~15‐fold more frequent than unification defects (bicornuate and didelphic uteri) (Chan et al. 2011), and have a continuous rather than a discrete distribution, suggesting that its growth is not tightly regulated.

If our hypothesis holds, how could short or long uterine horns develop? The identity of the respective segments of the Müllerian duct is determined by Hox genes, with the HOX9‐11 genes expressed differentially in the mesenchyme surrounding the developing oviducts, uterus, and cervix (Taylor et al. 1997; Du and Taylor 2015). These caudal HOX genes regulate cranio‐caudal patterning in the female urogenital tract (Benson et al. 1996; Gendron et al. 1997). Their expression is regulated by the local concentration of retinoic acid (Marshall et al. 1996) and sex‐steroidal hormones (Daftary and Taylor 2006). In the cranial Müllerian duct, retinoic acid is produced and sensed in the mesenchyme (Nakajima et al. 2016). Estrogen‐receptor expression is highest in the mesenchyme surrounding the uterine epithelium (Cunha et al. 2021), whereas androgen‐receptor expression is more prevalent in the mesenchyme surrounding the caudal part of the Müllerian tube (Drews et al. 2002). Throughout urogenital development, a relative deficiency of a functional HOX gene causes the expression of the 3′‐neighboring Hox gene to expand in caudal direction (Ma et al. 1998). This caudal extension in expression change is accompanied by a more caudal position of the uterotubal junction (Benson et al. 1996; Miller and Sassoon 1998) Although the effects of an increased expression of the caudal Hox genes on the morphogenesis of the female genital tract still have to be established, such experiments do show a re‐specification of vertebral identities (Kessel 1992).

### 4.4 Causes and Consequences of the Persistence of Completely Separate Müllerian Ducts

Uterus didelphys is an intriguing malformation with a prominent feature of a (near) complete lack of fusion between the parts of both Müllerian ducts that normally form the uterovaginal canal (Heinonen 2013). If a (near) complete separation exists, the rectum and bladder are connected by the so‐called rectovesical ligament. This fold separates the deepest part of the normally non‐septated pelvic peritoneum. In 7–8‐week‐old embryos, the pelvic part of the peritoneum extends to the pelvic floor, but subsequently obliterates in fetuses by fusion of the epithelial surfaces (Hikspoors et al. 2019). This peritoneal fusion process proceeds much like that in the upper abdomen, but peritoneal pouches may persist (Heald and Moran 1998). In patients with uterus didelphys, the left and right sides of the pelvic organs, including the Müllerian ducts, have not fused, which results in left‐ and right‐sided pelvic peritoneal cavities separated by the (rectovesical) septum (Figures 5 and 6).

### 4.5 The Caudal Ends of the Müllerian Ducts Do Not Fuse

Fusion of the caudal end of the Müllerian ducts is reproducibly absent. This absence of fusion of the caudal Müllerian ducts was described long ago by Kempermann (1931), Koff (1933), and more recently by Hashimoto (2003), but is notably absent in Bulmer's account (Bulmer 1957). Bulmer reported caudal fusion at 42 mm CRL [~9 weeks; (Bulmer 1957)], while Koff, Matejka, and we observed fusion at ~60 mm CRL [~10 weeks; (Koff 1933; Matejka 1959b)]. Koff claimed absence of fusion in 30% of embryos, while Hashimoto, like us, observed this unfused caudal end in all embryos studied. The bifid caudal end of the Müllerian ducts has a rather smooth surface and much thicker epithelium than the more cranial part of the Müllerian duct [Figure 4; (Kempermann 1931; Koff 1933; Witschi 1970)]. We named it the “head”, Koff the “solid tip,” and Vilas (Vilas 1932) and Matejka the bulbs (“Wülste”) of the uterovaginal canal. We did not adopt Koff's terminology, because a lumen remains identifiable until at least 11 weeks [Figure 7A; (Hülsman, Köhler, et al. 2025c; Matejka 1959a)] and, in somewhat modified form, until ~13 weeks [Figure 9B; (Hülsman, Köhler, et al. 2025c)]. Matejka identifies the caudal bifurcation of the uterovaginal lumen somewhat confusingly as the “vaginal septum” (Matejka 1959b). The caudal head or bulb of the uterovaginal canal produces a protrusion in the dorsal wall of the urogenital sinus that is known as the “Müllerian tubercle”. The Müllerian tubercle is largest at ~9 weeks (Koff 1933). Of interest, the adjacent caudal parts of the Wolffian ducts that correspond in position with the enlarged caudal end of the Müllerian ducts are also prominently enlarged. This feature made Koff believe, according to Drews (Mauch et al. 1985; Drews 2007), that they were part of the dorsal wall of the urogenital sinus and has led to much confusion about their lineage (see accompanying paper).

### 4.6 The Insertions of the Round Ligament of the Uterus (Gubernaculum)

We were unable to identify, among the ligaments that surround the ovary, the proper ovarian ligament in fetuses up to 12 weeks. We hypothesize, therefore, that the proper ovarian ligament is still part of the mesovarium. If so, the histological features of the round ligament and the mesovarium are very different. The fibromuscular phenotype is the distinguishing feature of the round ligament (Figures 12 and S6), whereas loose connective tissue characterizes the mesovarium and the suspensory ligament. Of note, the dominant histological feature of the fetal suspensory ligament of early female fetuses is the persistence of large parts of the caudal mesonephros, whereas only minor remnants of the mesonephros are usually found in adults. The gubernaculum attaches to the peri‐Wolffian mesenchyme at the tubo‐uterine junction [Figure 10C; fig. 3 in (van der Schoot 1996)] and is a convenient landmark to identify this junction.

The peritoneal evagination that extends around the gubernaculum in females is clinically known as the processus vaginalis or canal of Nuck, a Dutch anatomist (Sameshima et al. 2017). Most of the controversies about Nuck's canal regard its caudal attachment of the gubernaculum in the labia majora (classical view) or near the internal inguinal ligament [present view; (Ludwig 1993; van der Schoot 1996; Bouzada et al. 2017)], or in the mons pubis near the pubic tubercle [reported here; (Attah and Hutson 1991)]. The cause of these differences in interpretation can be partly attributed to a shortage in histological studies. In addition, the controversy about the caudal end of the round ligament may be due to the fanning out and mottling of the tissues that form the round ligament in its final course. In our most advanced fetus that allowed reconstruction of the canal of Nuck (12 weeks), its open connection with the peritoneal cavity is located ventrocaudal to the tubo‐uterine junction. From there, it courses laterally and then mainly caudally to enter its final extraperitoneal course. Some descriptions have it terminate between the rectus and internal oblique abdominal muscles to end at the internal inguinal ring (Ludwig 1993; van der Schoot 1996), whereas others have it breach the aponeurosis of the external oblique muscle before it finally fans out in the subcutaneous tissue of the mons pubis near the pubic tubercle [this study; (Attah and Hutson 1991)]. Muscle fibers of the oblique abdominal muscle appear also to become part of the encircling muscle fibers of the round ligament (Figure S6C,D) (Tanyel et al. 2005). Normally, the cranial part of Nuck's canal probably loses its patency in the late fetal period, but persisting patency has been reported (Kapur et al. 1998; Rees et al. 2017). Very little basic information is available about the timing of the peritoneal closure of the canal of Nuck, but at least one study put it at ~2 months after birth (Sameshima et al. 2017).

## 5 Conclusion

Between 5 and 6 weeks of development (Carnegie stages 15–18), the morphogenesis of Müllerian ducts (the establishment of the infundibular portion of the uterine tubes) proceeds in a Wolffian‐duct independent fashion. The subsequent caudal growth of the tubes is Wolffian‐duct dependent, although it is not entirely clear whether that also applies to the intrapelvic part, which becomes the uterus and vagina. A comparison of the early embryonic development of the intrapelvic part in mice and man revealed a high degree of similarity.

## Supporting information


**Figure S1:** ca70014‐sup‐0001‐FigureS1.jpg. **Protocol for generating 3D models of serial histological sections**. Histological sections are digitized (panel A). The digitized serial sections are uploaded to Amira to delineate the structures of interest (panel B1). The structure outlines are then stacked and aligned (panel B2). The stacked 3D outlines of Amira are next imported into Cinema4D to serve as reference (panel C1) for the creation of accurate 3D images (panel C2). See the Material & Methods section for more details.

**Figure S2:** ca70014‐sup‐0002‐FigureS2.jpg. **The Müllerian ducts enter the lesser pelvis and fuse at CS23** (embryo S4141). In panel A an overview section of the two Müllerian ducts meeting in the midline. Panel B shows a close‐up of this event. The basement membrane of the facing sides of the ducts is less well developed than that of the free sides. GC: genital cord; MD: fusing Müllerian ducts; Re: rectum; UGS: urogenital sinus; UVC: uterovaginal canal; VP: vaginal plate; WD: Wolffian duct. Bars: A: 0.2 mm; B: 0.1 mm.

**Figure S3:** ca70014‐sup‐0003‐FigureS3.jpg. **The development of the genital cord between 8 and 12 weeks of development** (S48, S4141 and S2383). At 8 weeks the genital cord (GC) is still a homogeneous cylindrical mass of dense mesenchyme that surrounds the genital ducts (panels A and B). Panel A shows a male, and panel B a female embryo. Note the septum in the uterovaginal canal in panels A and the difference in diameter of the Wolffian ducts in panels A and B. Laterally, the genital cord is flanked by the autonomic hypogastric ganglia. At 12 weeks (panel C) the outside of the genital cord has become denser and separates the genital ducts from their environment. GC: genital cord; MD: Müllerian duct; Re: rectum; UGS: urogenital sinus; WD: Wolffian duct. Bars: 0.2 mm.

**Figure S4:** ca70014‐sup‐0004‐FigureS4.jpg. **Histology of the gubernaculum in the 7th week** (S2025 and Goe_1947_07_02). The gubernaculum tethers the caudal mesonephros and the genital ducts to the lateral body wall. Note that smooth muscle is primarily found in its caudal sector. G: gubernaculum; M: mesonephros; MD: Müllerian duct; Re: rectum; WD: Wolffian duct. Bars: 0.2 mm.

**Figure S5:** ca70014‐sup‐0005‐FigureS5.jpg. **Histology of the gubernaculum and the canal of Nuck in the 8th and 9th weeks (**S9226 and S89). Panels A and B are from the same 8‐week‐old embryo. Panel C is a histological section of the gubernaculum at 9 weeks. The arrows pointing towards the canal of Nuck. Asterisk: extraperitoneal part of gubernaculum breaching the ventral body wall. G: gubernaculum; MD: Müllerian duct; Ov: ovary; Re: rectum; WD: Wolffian duct. Bars: 0.5 mm.

**Figure S6:** ca70014‐sup‐0006‐FigureS6.jpg. **The course and histology of the gubernaculum at 12 weeks** (fetus S2383). The section is taken at the level of the uterus fundus at which the uterine tube connects to it. The arrows indicate the gubernaculum at different locations in the lesser pelvis. The asterisks show the histology of the gubernaculum at the caudal end of the canal of Nuck (panel C) and its subsequent extraperitoneal course (panels D). B: bladder; Re: rectum; Ut uterus. Bars: 1 mm.

**Figure S7:** ca70014‐sup‐0007‐FigureS7.jpg. **Histology of the uterine tubes at 12 weeks of development** (fetus S2383). In panel A an overview of the uterus and uterine tubes is shown, with a higher magnification in panel A1. Note the folding of the epithelium and the appearance of a smooth‐muscle layer that surrounds the uterine tubes (black arrowheads). Bars: 0.5 mm.
**3D‐PDF instructions**.
To view the interactive 3D‐PDFs in their full potential you need to download the 3D‐PDFs to your computer (a 3D‐PDF can be opened on any computer as long as it contains the Adobe PDF reader). To activate the 3D‐PDF you need to click on the model. A toolbar appears at the top of the screen. Under options, you must state that you trust this document. If you then click on the model (version 24 and later: with the right mouse button), a toolbar appears on the left side of your screen (on the right side in version 24 and later) that includes the option “model tree.” The model tree displays a list of structures in the upper box and preset viewing options in the lower box. The list of visible structures can be modified by marking or unmarking a structure. We advise to start with a basal configuration that contains a few structures only and add structures to this simple configuration rather than the other way around: “dress, do not undress.” To manipulate the reconstruction, press the left mouse button to rotate it, the scroll button to zoom in or out, and the left and right mouse buttons simultaneously to move the embryo across the screen. The color code is identical in all figures, and all structures are listed by the same name and relative position in the “model tree.” The edges of the scale cube are 1 mm.

**Figure S8:** ca70014‐sup‐0008‐FigureS8.pdf. **Interactive 3D‐PDF of pleuroperitoneal membrane of a ~5‐week‐old human embryo** (CS15; S721). In the pdf the inferior surface of the parietal pleura (purple), the peritoneal surface of the pleuroperitoneal membrane (green), and the craniodorsal wall of the peritoneum (light orange) are visible.

**Figure S9:** ca70014‐sup‐0009‐FigureS9.pdf. **Interactive 3D‐pdf of the urogenital region of a ~6‐week‐old human embryo** (CS18; S4430). The Müllerian duct invaginates into the underlying mesenchyme and the first part of the tube forms alongside the Wolffian duct.

**Figure S10:** ca70014‐sup‐0010‐FigureS10.pdf. **Interactive 3D‐pdf of the urogenital region of a ~7‐week‐old human embryo** (CS20; S2025). The Müllerian duct elongates caudally in close proximity to the Wolffian duct.

**Figure S11:** ca70014‐sup‐0011‐FigureS11.pdf. **Interactive 3D‐pdf of the urogenital region of a ~7‐week‐old human embryo** (CS21; S4090). The Müllerian duct has almost reached the midline.

**Figure S12:** ca70014‐sup‐0012‐FigureS12.pdf. **Interactive 3D‐pdf of the urogenital region of a ~7.5‐week‐old human embryo** (CS22; S983). At this stage, the intrapelvic part of the Müllerian ducts fuses, but the caudal end remains unfused. The Müllerian duct had also reached the urogenital sinus.

**Figure S13:** ca70014‐sup‐0013‐FigureS13.pdf. **Interactive 3D‐pdf of the urogenital region of a ~8‐week‐old human embryo** (CS23; S48). The lumen of the Müllerian duct has not yet completely fused into a single lumen.

**Figure S14:** ca70014‐sup‐0014‐FigureS14.pdf. **Interactive 3D‐pdf of the urogenital region of a ~8‐week‐old human embryo** (CS23; S4141). The lumen of the Müllerian duct is completely fused except for its most caudal part.

**Figure S15:** ca70014‐sup‐0015‐FigureS15.pdf. **Interactive 3D‐pdf of the urogenital region of a ~9‐week‐old human embryo** (S89).

**Figure S16:** ca70014‐sup‐0016‐FigureS16.pdf. **Interactive 3D‐pdf of the urogenital region of a ~10‐week‐old human embryo** (S4908).

**Figure S17:** ca70014‐sup‐0017‐FigureS17.pdf. **Interactive 3D‐pdf of the urogenital region of a ~10‐week‐old human embryo** (S1744).

**Figure S18:** ca70014‐sup‐0018‐FigureS18.pdf. **Interactive 3D‐pdf of the urogenital region of a ~11‐week‐old human embryo** (S1743).

**Figure S19:** ca70014‐sup‐0019‐FigureS19.pdf. **Interactive 3D‐pdf of the urogenital region of a ~12‐week‐old human embryo** (S2383).

**Figure S20:** ca70014‐sup‐0020‐FigureS20.pdf. **Interactive 3D‐pdf of the urogenital region of a ~13‐week‐old human embryo** (S2212). The cervix has a distinctive rugged surface.

**Figure S21:** ca70014‐sup‐0021‐FigureS21.pdf. **Interactive 3D‐pdf of the urogenital region of a ~15‐week‐old human embryo** (S2392). 30%–35% of the vagina is now positioned caudal from the pubococcygeal line.

## Data Availability

The data that support the findings of this study are available from the corresponding author upon reasonable request.
