# Supplementary figures and images for: The Development of the Human Female Reproductive Tract. Part 1: Uterine Tube and Uterus
Source: Clin Anat. 2025 Oct 6;39(1):92–111. doi: 10.1002/ca.70014 (PMC12747624; doi:10.1002/ca.70014)

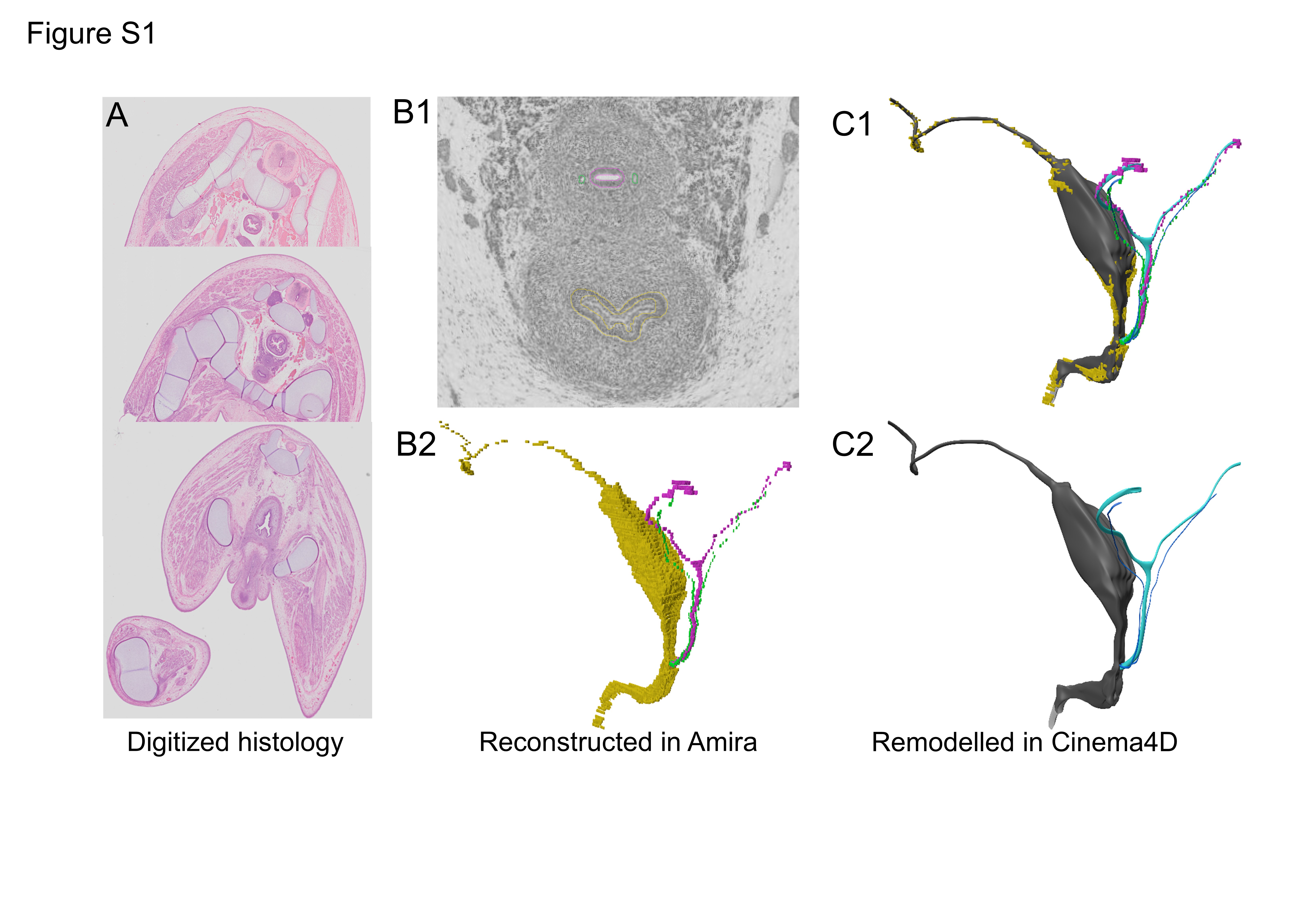

Supplement: Supplementary file 1 — Figure S1: ca70014‐sup‐0001‐FigureS1.jpg. Protocol for generating 3D models of serial histological sections. Histological sections are digitized (panel A). The digitized serial sections are uploaded to Amira to delineate the structures of interest (panel B1). The structure outlines are then stacked and aligned (panel B2). The stacked 3D outlines of Amira are next imported into Cinema4D to serve as reference (panel C1) for the creation of accurate 3D images (panel C2). See the Material & Methods section for more details. [file CA-39-92-s018.jpg]

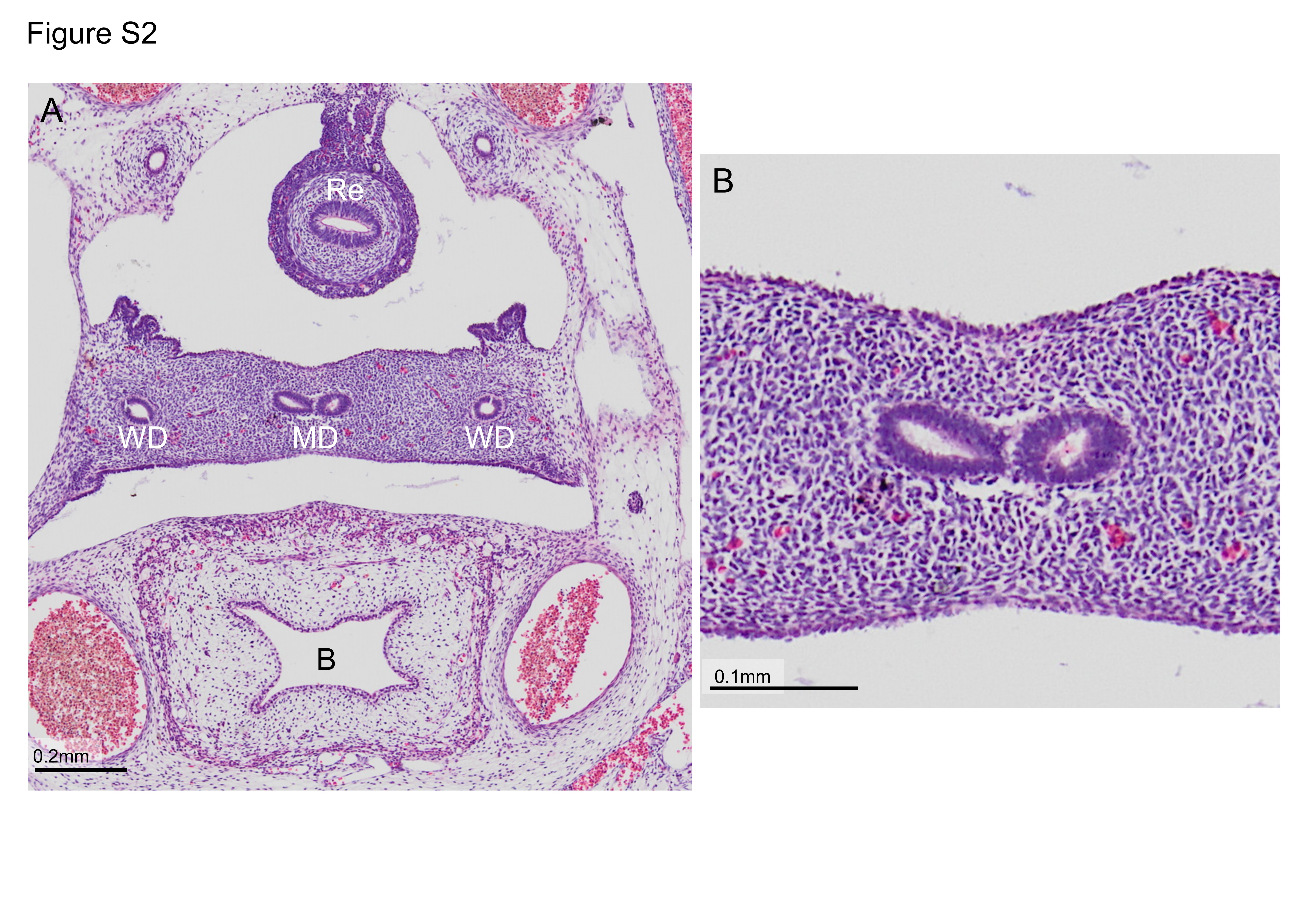

Supplement: Supplementary file 2 — Figure S2: ca70014‐sup‐0002‐FigureS2.jpg. The Müllerian ducts enter the lesser pelvis and fuse at CS23 (embryo S4141). In panel A an overview section of the two Müllerian ducts meeting in the midline. Panel B shows a close‐up of this event. The basement membrane of the facing sides of the ducts is less well developed than that of the free sides. GC: genital cord; MD: fusing Müllerian ducts; Re: rectum; UGS: urogenital sinus; UVC: uterovaginal canal; VP: vaginal plate; WD: Wolffian duct. Bars: A: 0.2 mm; B: 0.1 mm. [file CA-39-92-s019.jpg]

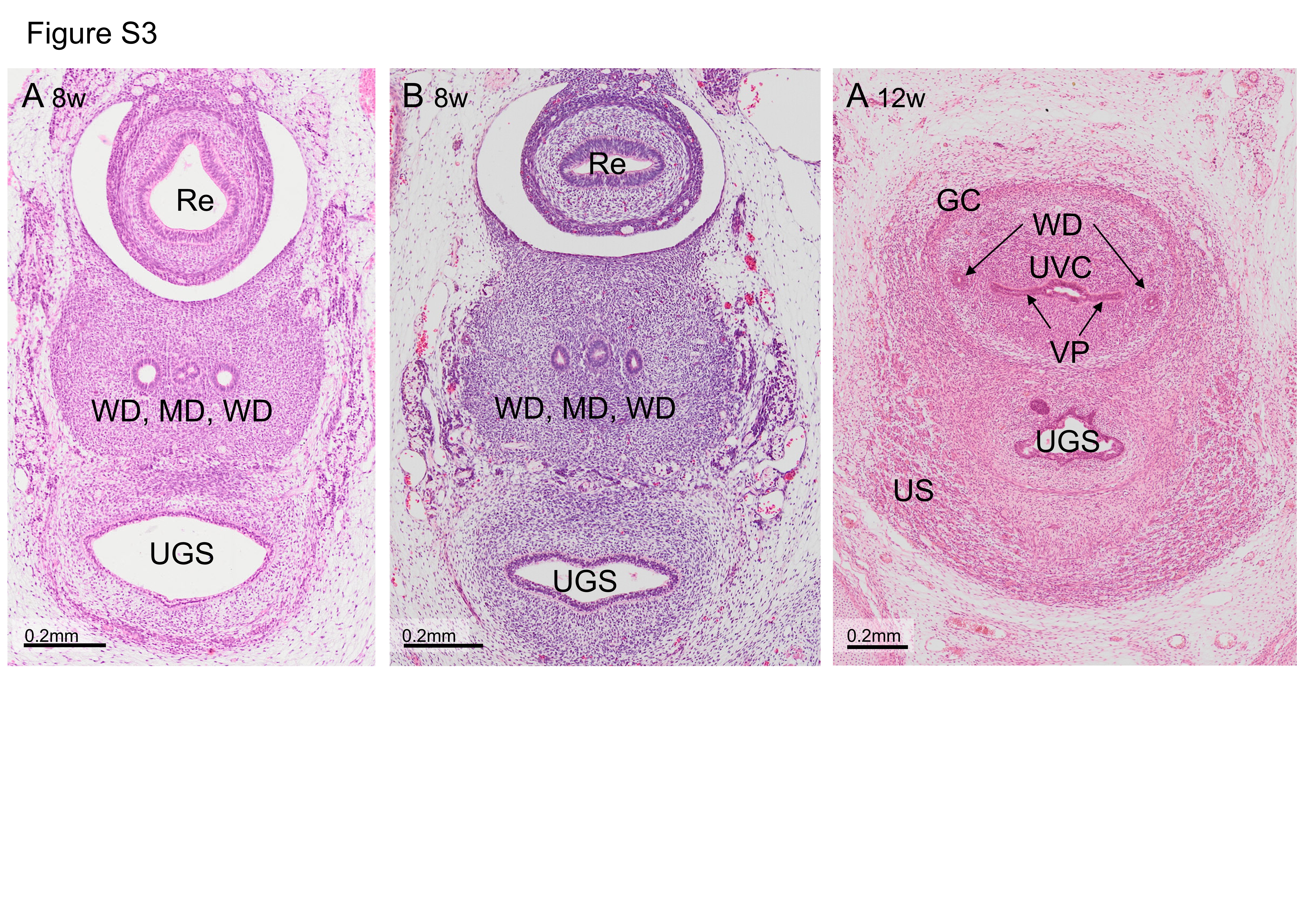

Supplement: Supplementary file 3 — Figure S3: ca70014‐sup‐0003‐FigureS3.jpg. The development of the genital cord between 8 and 12 weeks of development (S48, S4141 and S2383). At 8 weeks the genital cord (GC) is still a homogeneous cylindrical mass of dense mesenchyme that surrounds the genital ducts (panels A and B). Panel A shows a male, and panel B a female embryo. Note the septum in the uterovaginal canal in panels A and the difference in diameter of the Wolffian ducts in panels A and B. Laterally, the genital cord is flanked by the autonomic hypogastric ganglia. At 12 weeks (panel C) the outside of the genital cord has become denser and separates the genital ducts from their environment. GC: genital cord; MD: Müllerian duct; Re: rectum; UGS: urogenital sinus; WD: Wolffian duct. Bars: 0.2 mm. [file CA-39-92-s003.jpg]

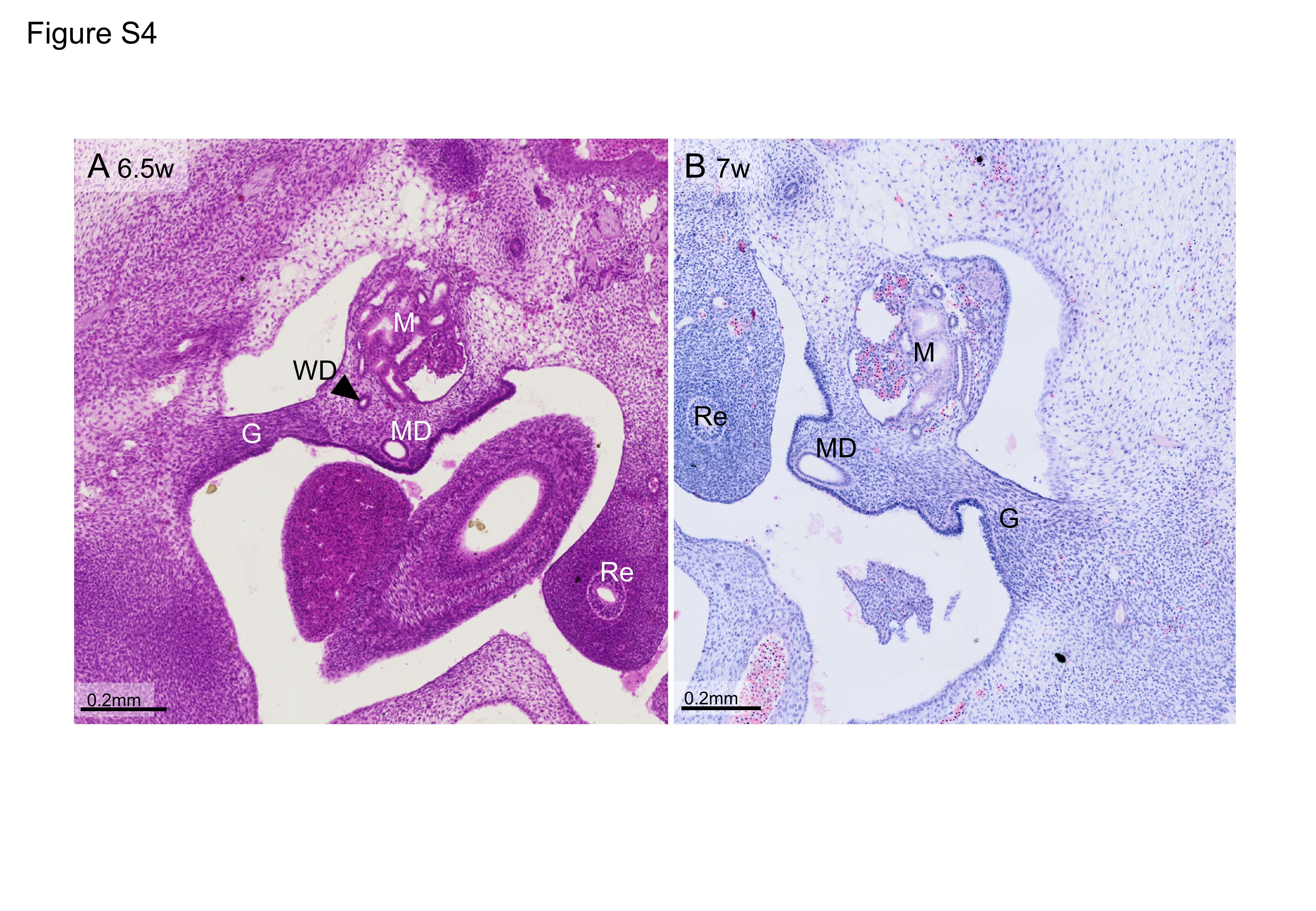

Supplement: Supplementary file 4 — Figure S4: ca70014‐sup‐0004‐FigureS4.jpg. Histology of the gubernaculum in the 7th week (S2025 and Goe_1947_07_02). The gubernaculum tethers the caudal mesonephros and the genital ducts to the lateral body wall. Note that smooth muscle is primarily found in its caudal sector. G: gubernaculum; M: mesonephros; MD: Müllerian duct; Re: rectum; WD: Wolffian duct. Bars: 0.2 mm. [file CA-39-92-s013.jpg]

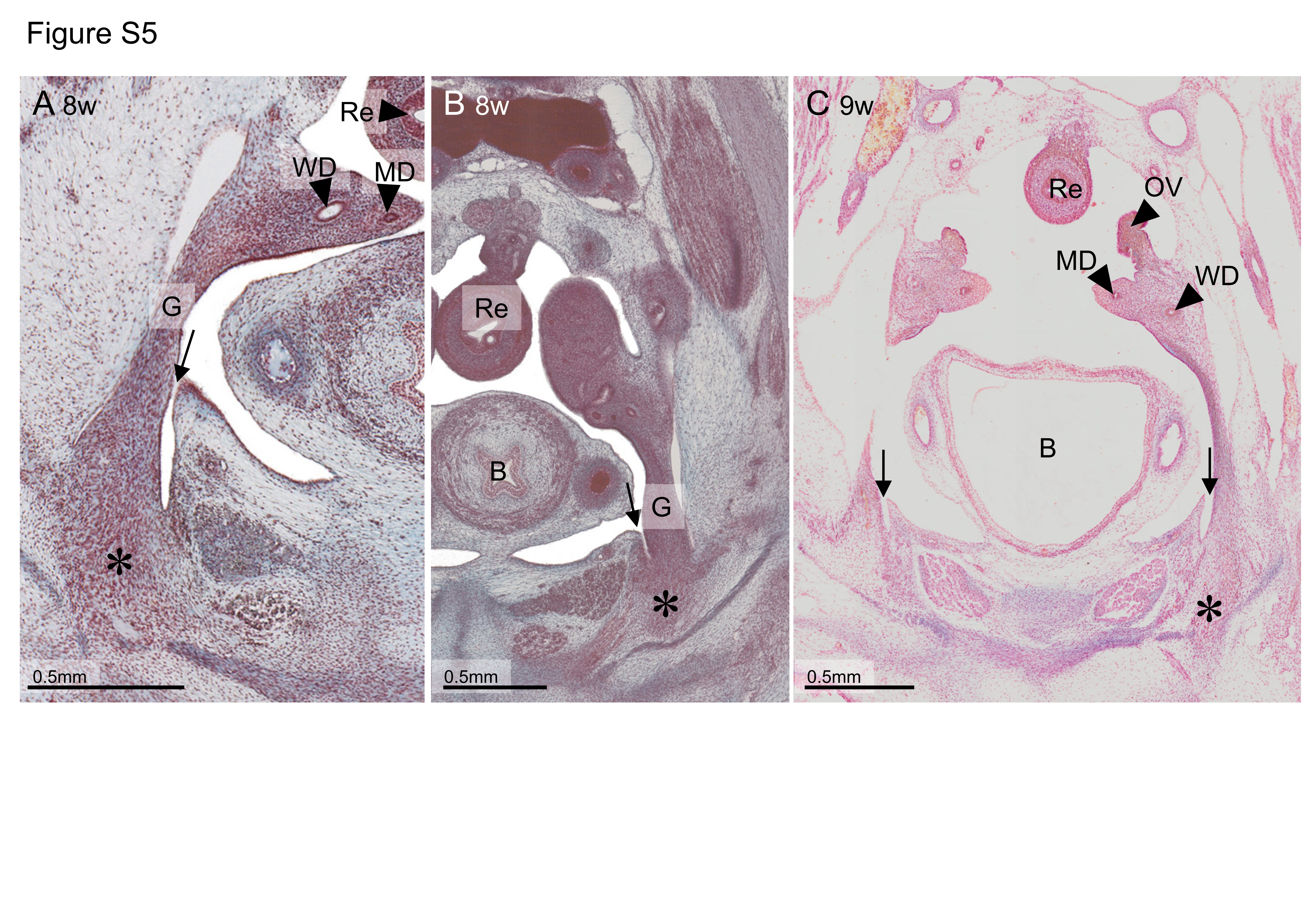

Supplement: Supplementary file 5 — Figure S5: ca70014‐sup‐0005‐FigureS5.jpg. Histology of the gubernaculum and the canal of Nuck in the 8th and 9th weeks (S9226 and S89). Panels A and B are from the same 8‐week‐old embryo. Panel C is a histological section of the gubernaculum at 9 weeks. The arrows pointing towards the canal of Nuck. Asterisk: extraperitoneal part of gubernaculum breaching the ventral body wall. G: gubernaculum; MD: Müllerian duct; Ov: ovary; Re: rectum; WD: Wolffian duct. Bars: 0.5 mm. [file CA-39-92-s006.jpg]

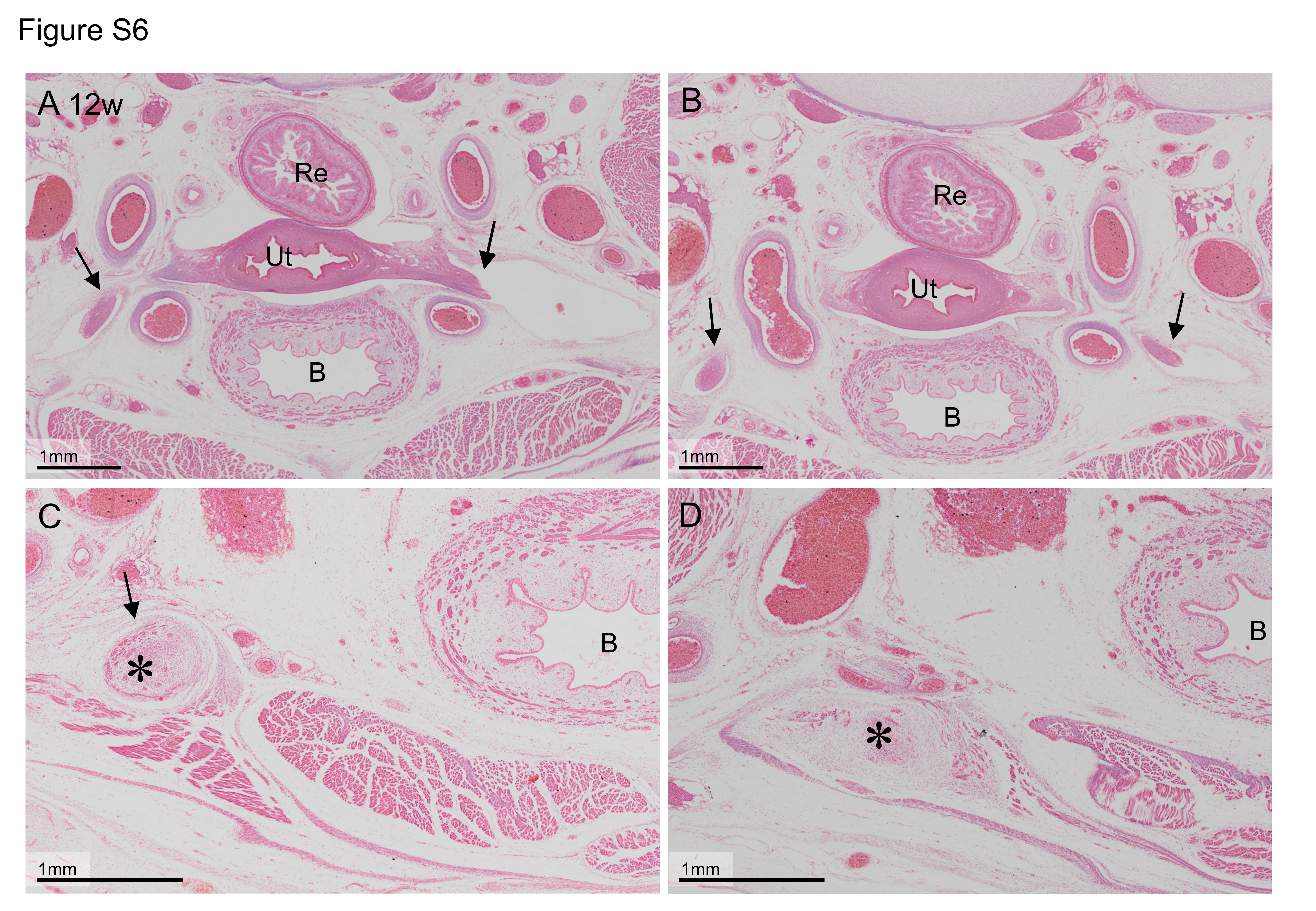

Supplement: Supplementary file 6 — Figure S6: ca70014‐sup‐0006‐FigureS6.jpg. The course and histology of the gubernaculum at 12 weeks (fetus S2383). The section is taken at the level of the uterus fundus at which the uterine tube connects to it. The arrows indicate the gubernaculum at different locations in the lesser pelvis. The asterisks show the histology of the gubernaculum at the caudal end of the canal of Nuck (panel C) and its subsequent extraperitoneal course (panels D). B: bladder; Re: rectum; Ut uterus. Bars: 1 mm. [file CA-39-92-s007.jpg]

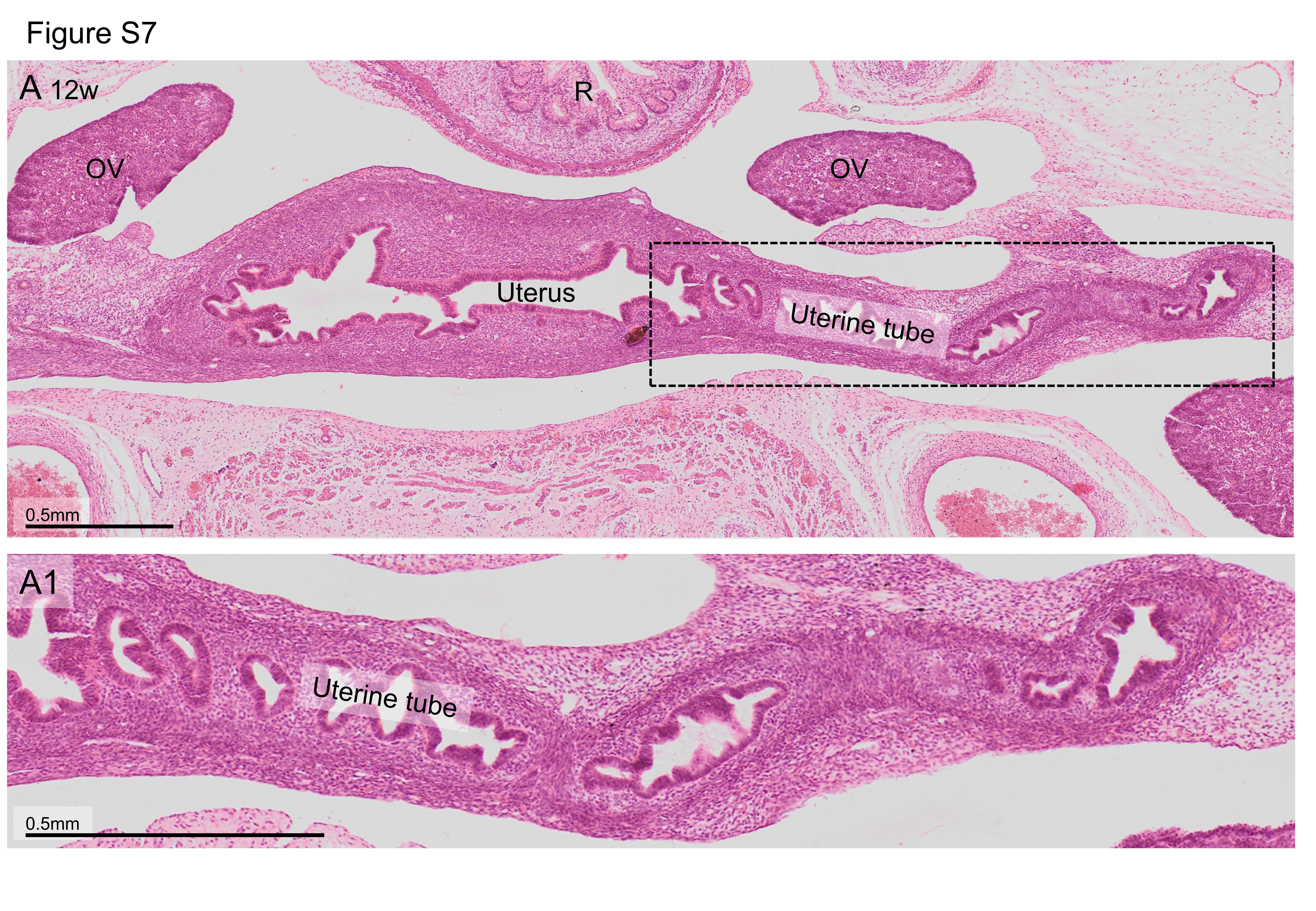

Supplement: Supplementary file 7 — Figure S7: ca70014‐sup‐0007‐FigureS7.jpg. Histology of the uterine tubes at 12 weeks of development (fetus S2383). In panel A an overview of the uterus and uterine tubes is shown, with a higher magnification in panel A1. Note the folding of the epithelium and the appearance of a smooth‐muscle layer that surrounds the uterine tubes (black arrowheads). Bars: 0.5 mm. 3D‐PDF instructions. To view the interactive 3D‐PDFs in their full potential you need to download the 3D‐PDFs to your computer (a 3D‐PDF can be opened on any computer as long as it contains the Adobe PDF reader). To activate the 3D‐PDF you need to click on the model. A toolbar appears at the top of the screen. Under options, you must state that you trust this document. If you then click on the model (version 24 and later: with the right mouse button), a toolbar appears on the left side of your screen (on the right side in version 24 and later) that includes the option “model tree.” The model tree displays a list of structures in the upper box and preset viewing options in the lower box. The list of visible structures can be modified by marking or unmarking a structure. We advise to start with a basal configuration that contains a few structures only and add structures to this simple configuration rather than the other way around: “dress, do not undress.” To manipulate the reconstruction, press the left mouse button to rotate it, the scroll button to zoom in or out, and the left and right mouse buttons simultaneously to move the embryo across the screen. The color code is identical in all figures, and all structures are listed by the same name and relative position in the “model tree.” The edges of the scale cube are 1 mm. [file CA-39-92-s014.jpg]

CS15

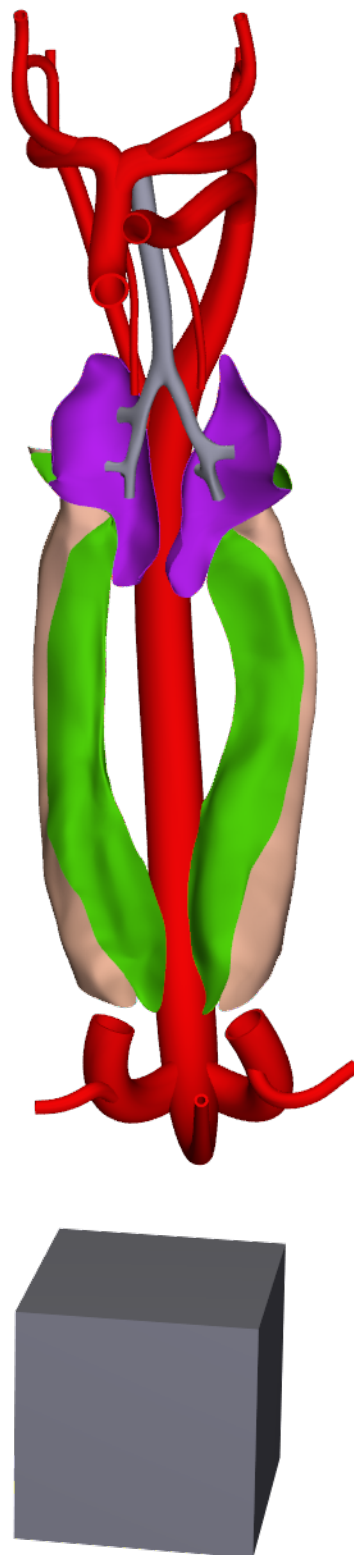

Supplement: Supplementary file 8 — Figure S8: ca70014‐sup‐0008‐FigureS8.pdf. Interactive 3D‐PDF of pleuroperitoneal membrane of a ~5‐week‐old human embryo (CS15; S721). In the pdf the inferior surface of the parietal pleura (purple), the peritoneal surface of the pleuroperitoneal membrane (green), and the craniodorsal wall of the peritoneum (light orange) are visible. [file CA-39-92-s010.pdf]

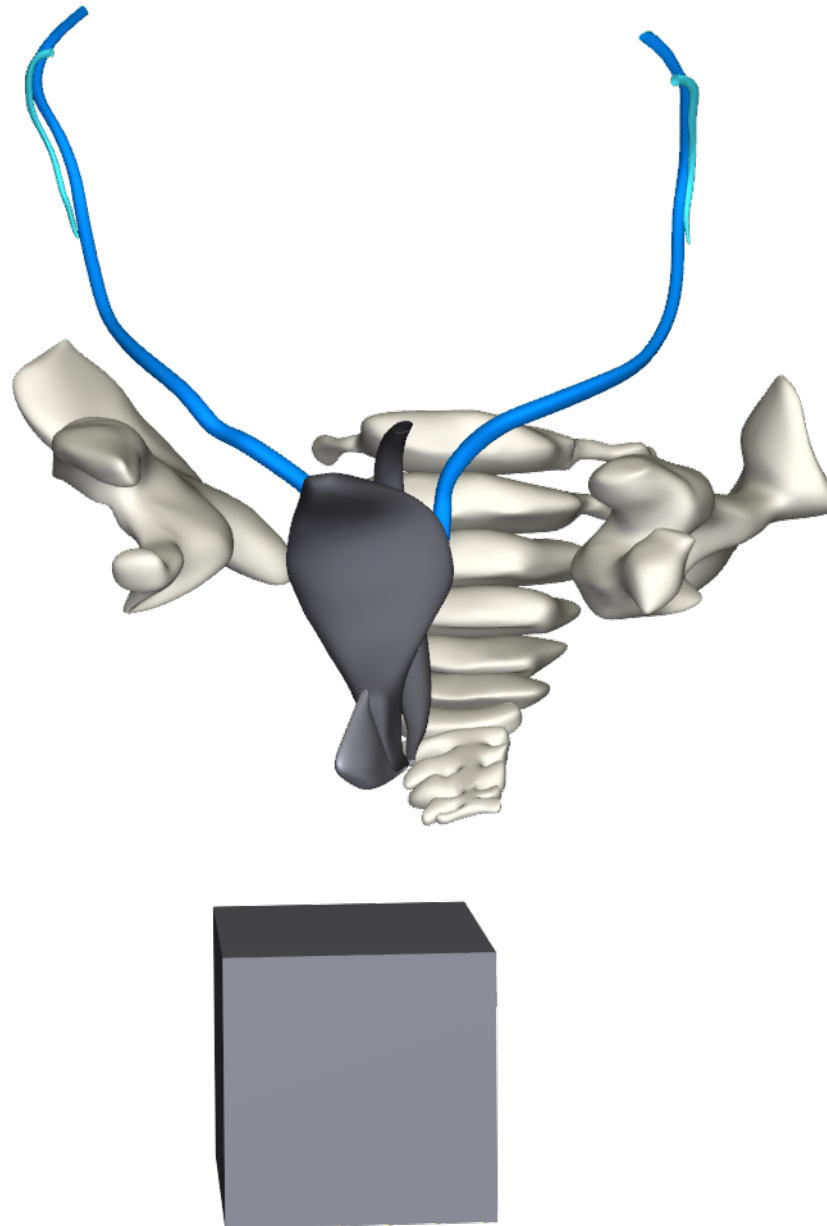

Supplement: Supplementary file 9 — Figure S9: ca70014‐sup‐0009‐FigureS9.pdf. Interactive 3D‐pdf of the urogenital region of a ~6‐week‐old human embryo (CS18; S4430). The Müllerian duct invaginates into the underlying mesenchyme and the first part of the tube forms alongside the Wolffian duct. [file CA-39-92-s012.pdf]

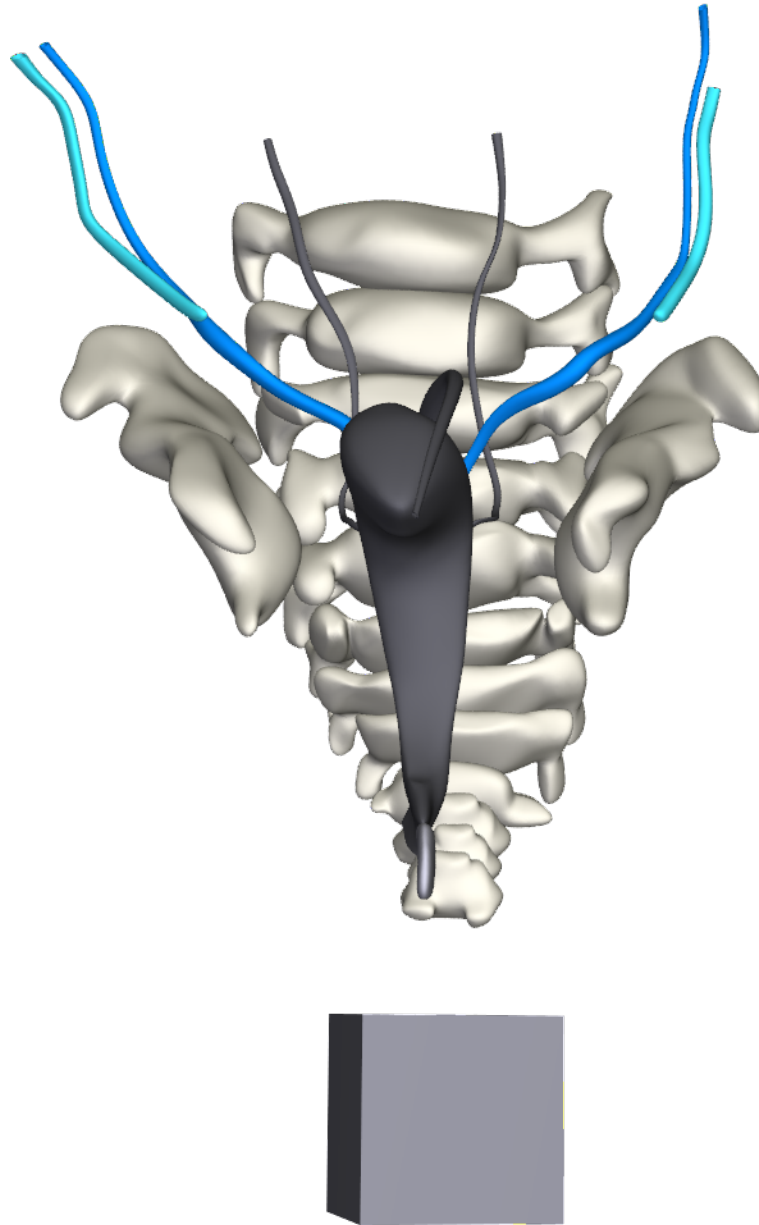

Supplement: Supplementary file 10 — Figure S10: ca70014‐sup‐0010‐FigureS10.pdf. Interactive 3D‐pdf of the urogenital region of a ~7‐week‐old human embryo (CS20; S2025). The Müllerian duct elongates caudally in close proximity to the Wolffian duct. [file CA-39-92-s020.pdf]

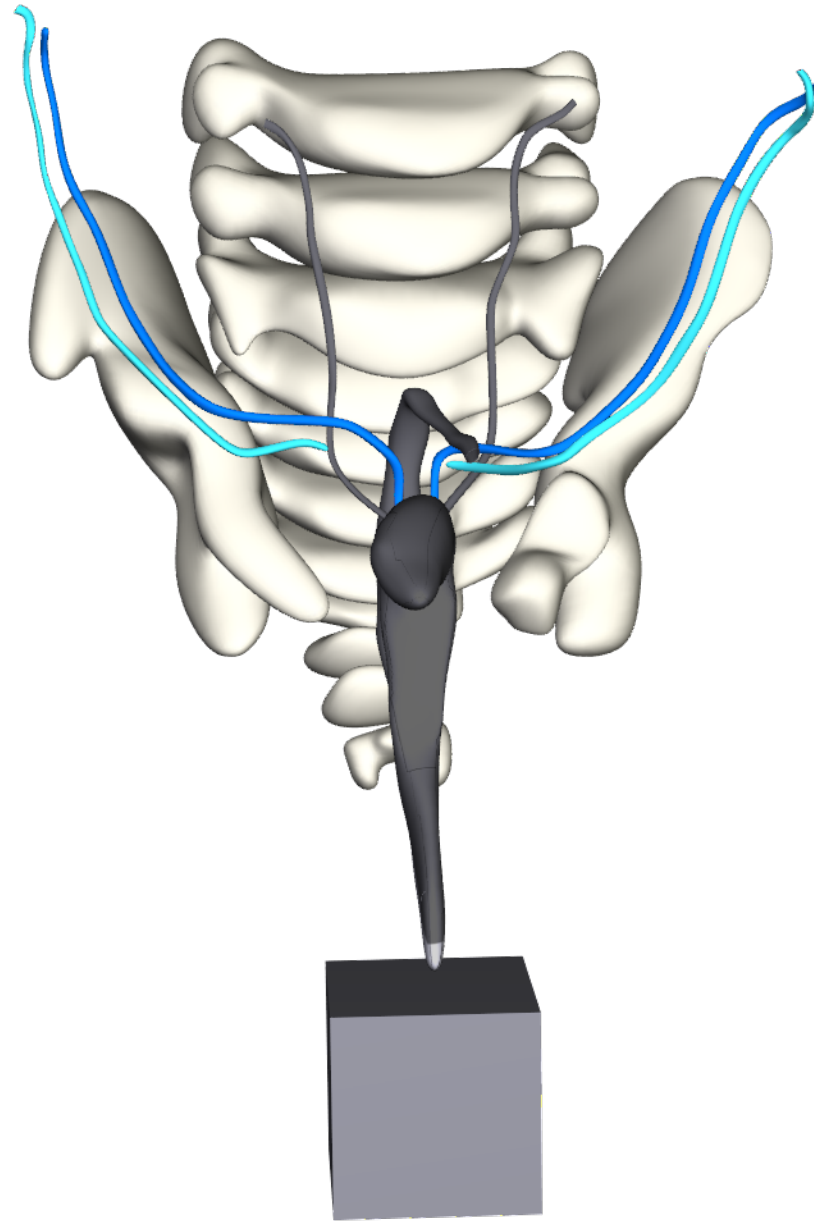

Supplement: Supplementary file 11 — Figure S11: ca70014‐sup‐0011‐FigureS11.pdf. Interactive 3D‐pdf of the urogenital region of a ~7‐week‐old human embryo (CS21; S4090). The Müllerian duct has almost reached the midline. [file CA-39-92-s004.pdf]

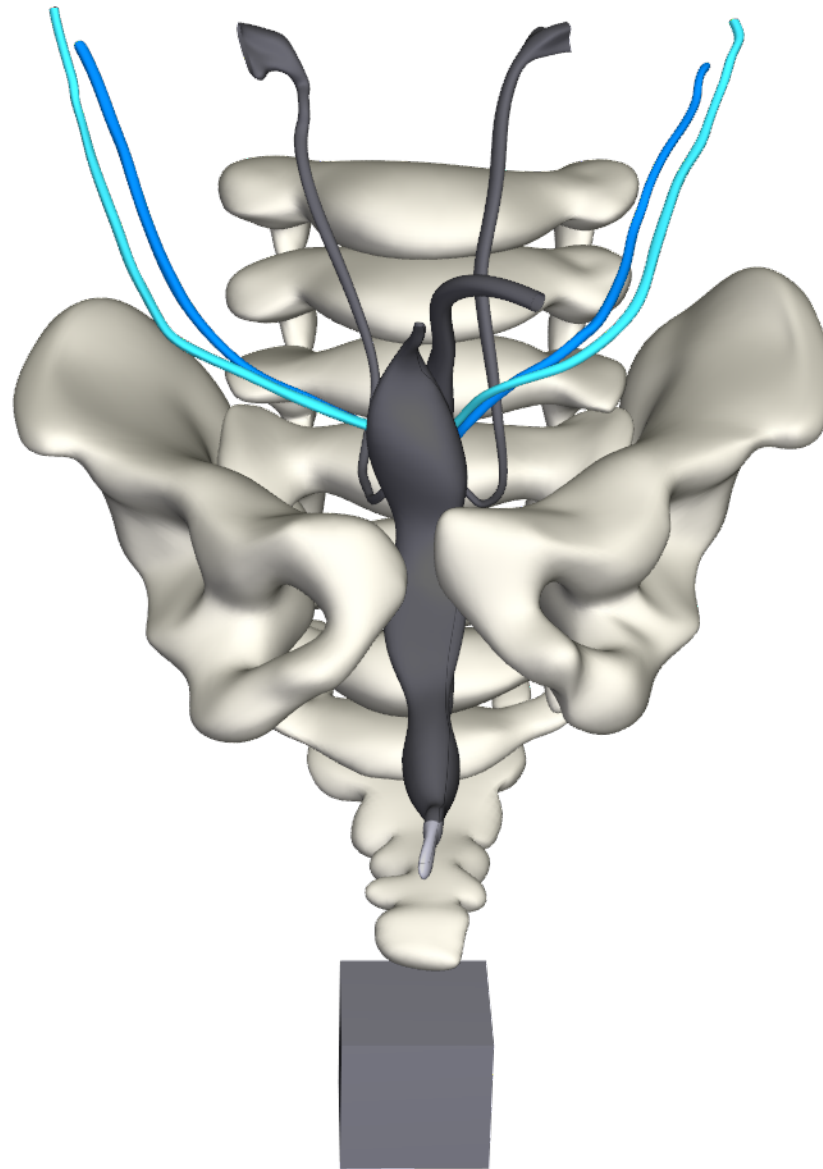

Supplement: Supplementary file 12 — Figure S12: ca70014‐sup‐0012‐FigureS12.pdf. Interactive 3D‐pdf of the urogenital region of a ~7.5‐week‐old human embryo (CS22; S983). At this stage, the intrapelvic part of the Müllerian ducts fuses, but the caudal end remains unfused. The Müllerian duct had also reached the urogenital sinus. [file CA-39-92-s011.pdf]

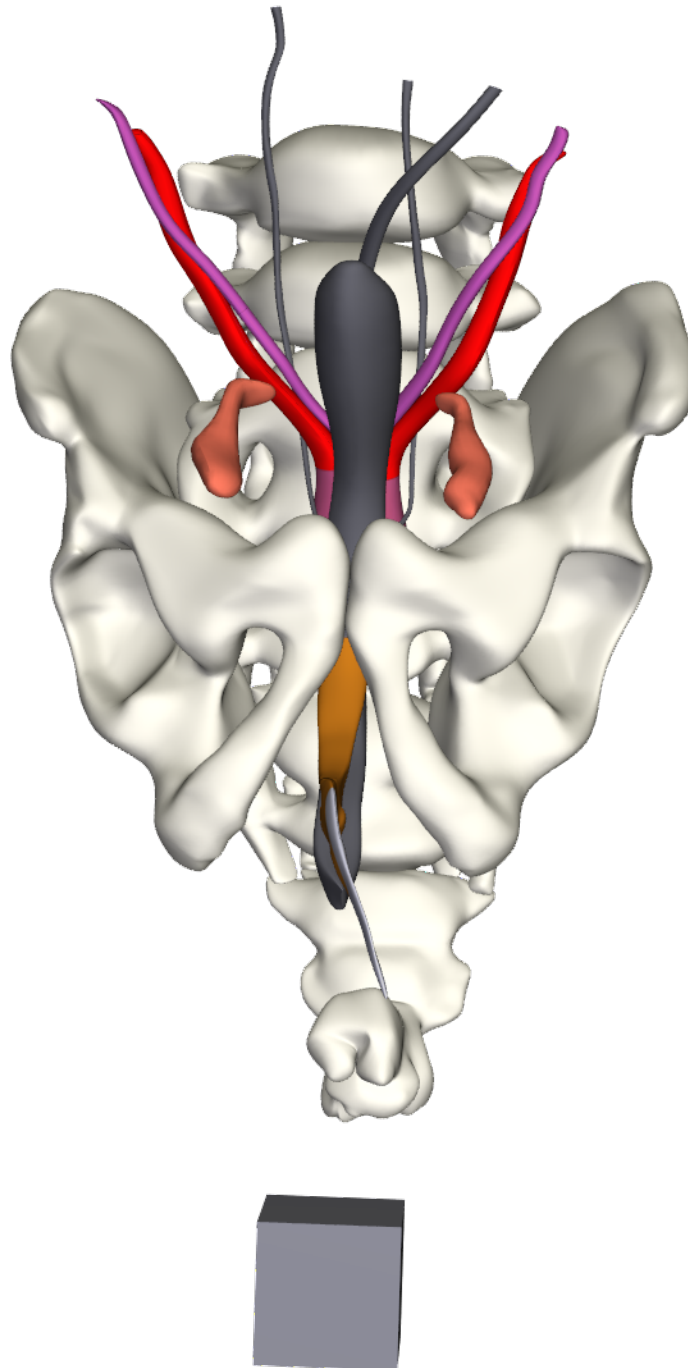

Supplement: Supplementary file 13 — Figure S13: ca70014‐sup‐0013‐FigureS13.pdf. Interactive 3D‐pdf of the urogenital region of a ~8‐week‐old human embryo (CS23; S48). The lumen of the Müllerian duct has not yet completely fused into a single lumen. [file CA-39-92-s015.pdf]

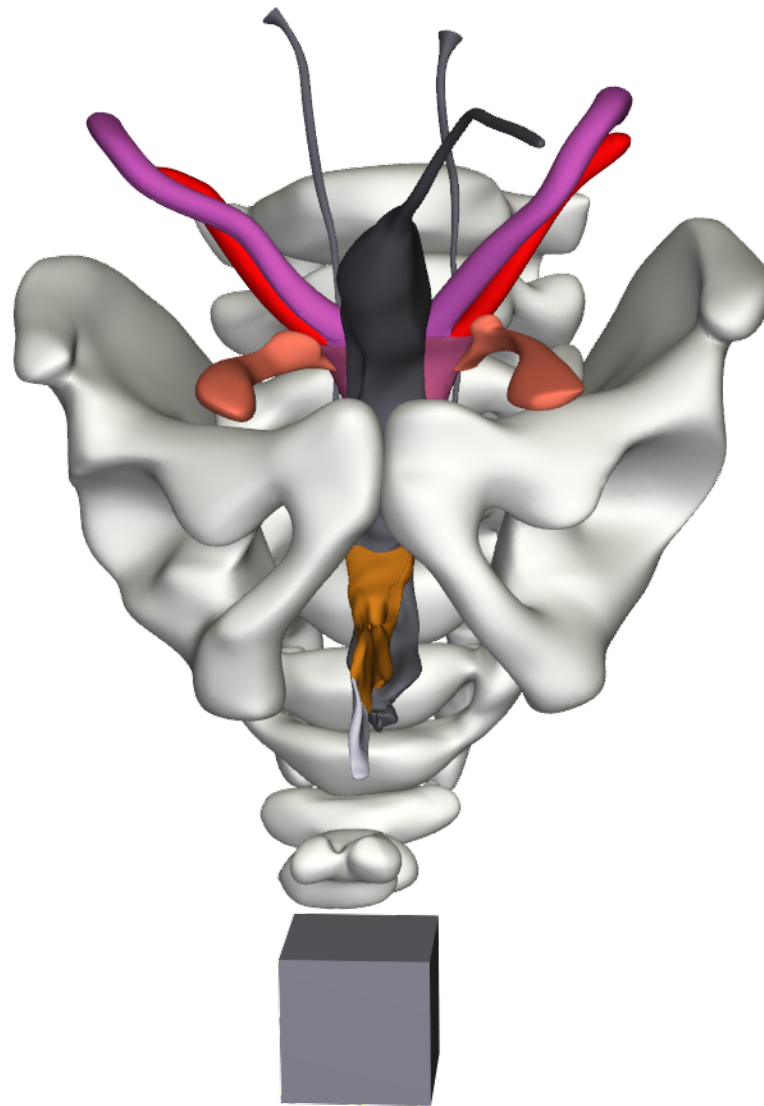

Supplement: Supplementary file 14 — Figure S14: ca70014‐sup‐0014‐FigureS14.pdf. Interactive 3D‐pdf of the urogenital region of a ~8‐week‐old human embryo (CS23; S4141). The lumen of the Müllerian duct is completely fused except for its most caudal part. [file CA-39-92-s005.pdf]

9 Weeks (S89)

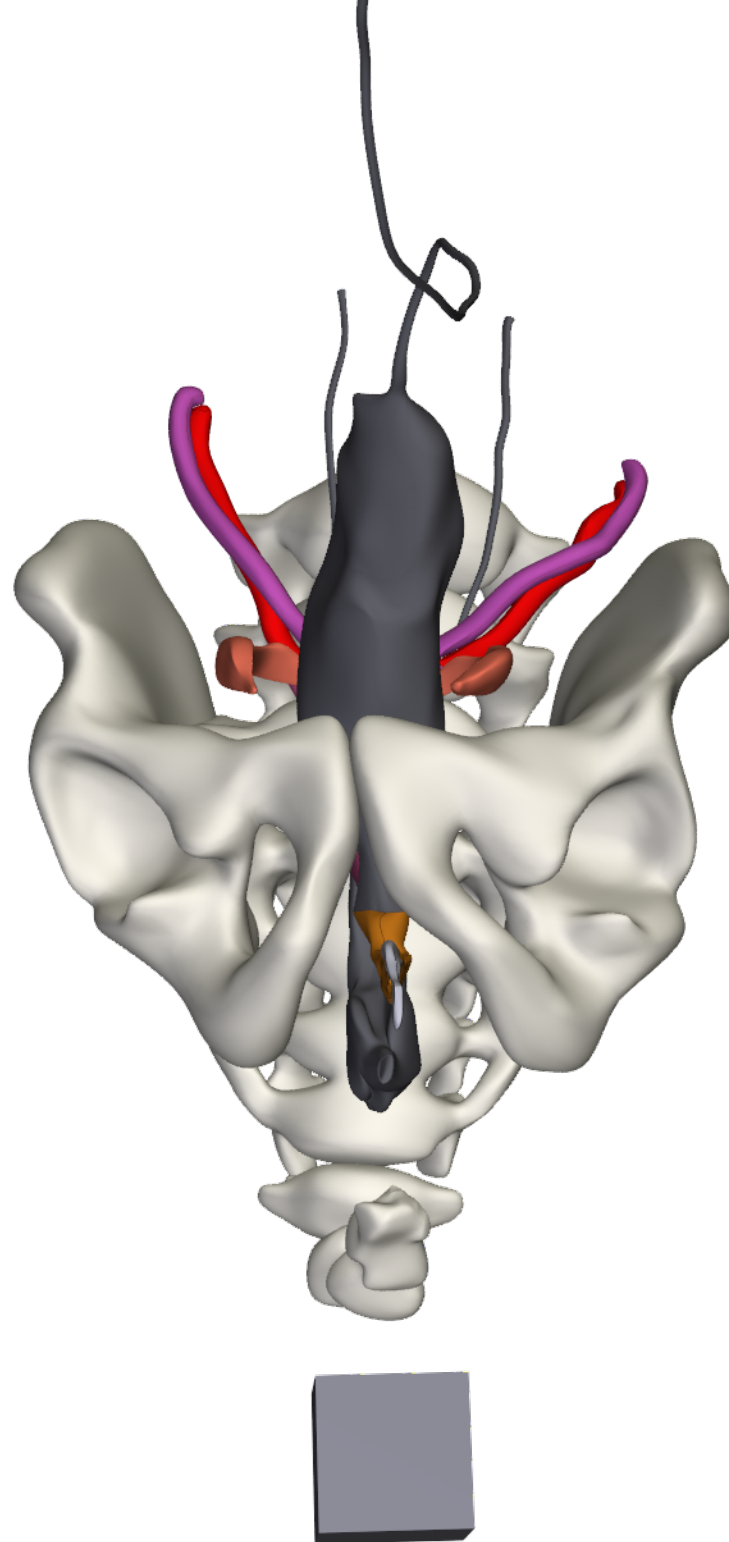

Supplement: Supplementary file 15 — Figure S15: ca70014‐sup‐0015‐FigureS15.pdf. Interactive 3D‐pdf of the urogenital region of a ~9‐week‐old human embryo (S89). [file CA-39-92-s009.pdf]

10 Weeks (S4908)

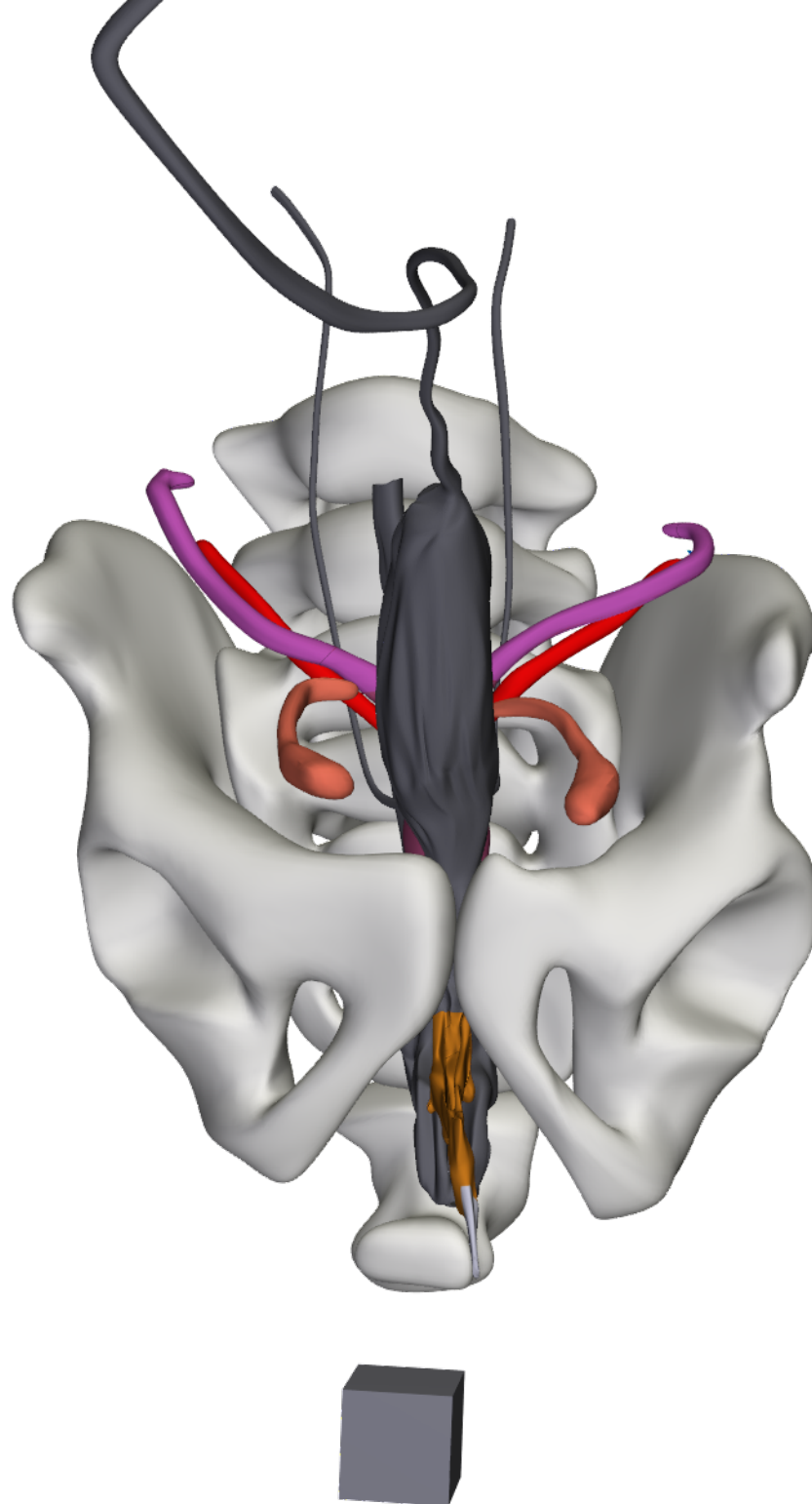

Supplement: Supplementary file 16 — Figure S16: ca70014‐sup‐0016‐FigureS16.pdf. Interactive 3D‐pdf of the urogenital region of a ~10‐week‐old human embryo (S4908). [file CA-39-92-s001.pdf]

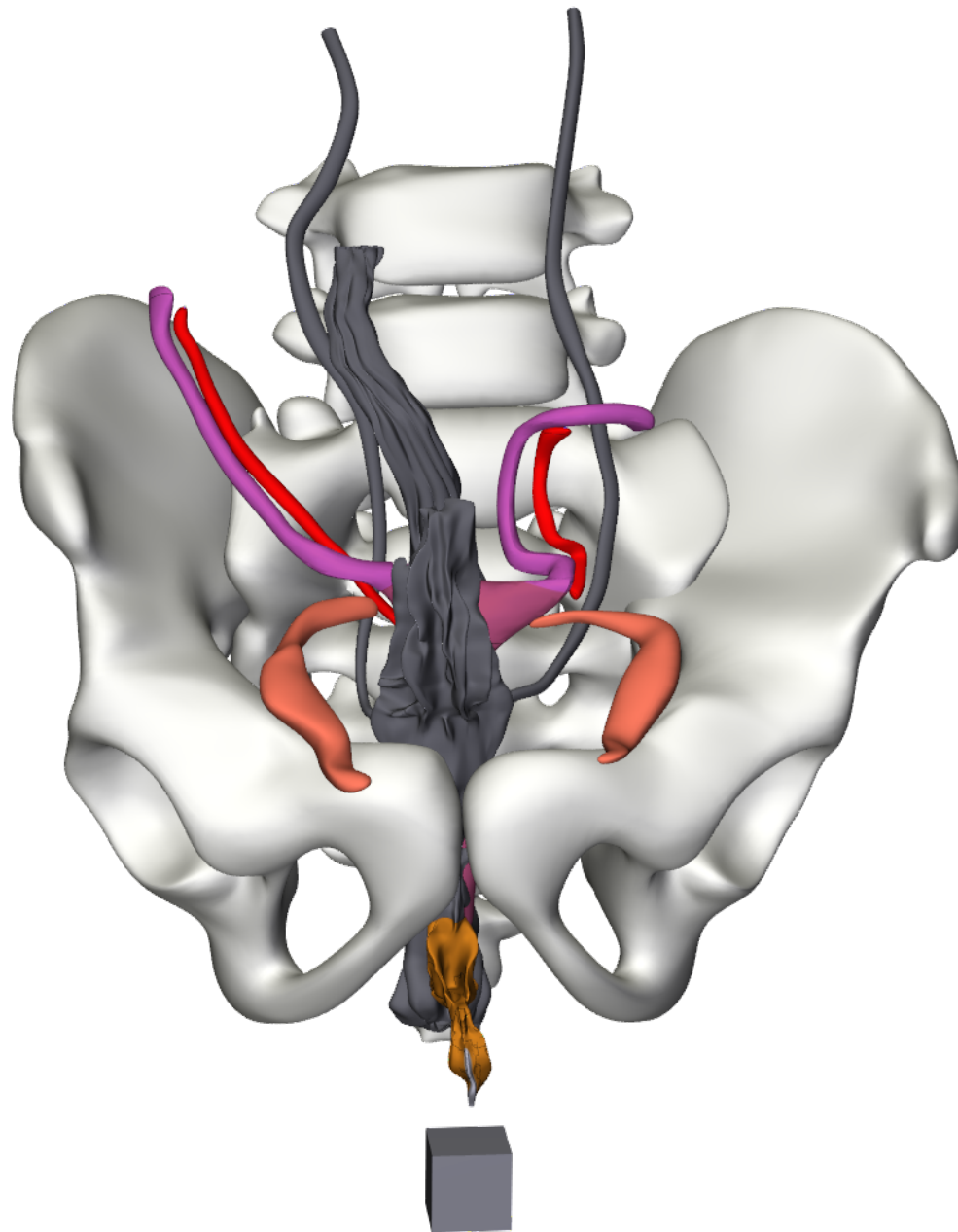

Supplement: Supplementary file 17 — Figure S17: ca70014‐sup‐0017‐FigureS17.pdf. Interactive 3D‐pdf of the urogenital region of a ~10‐week‐old human embryo (S1744). [file CA-39-92-s016.pdf]

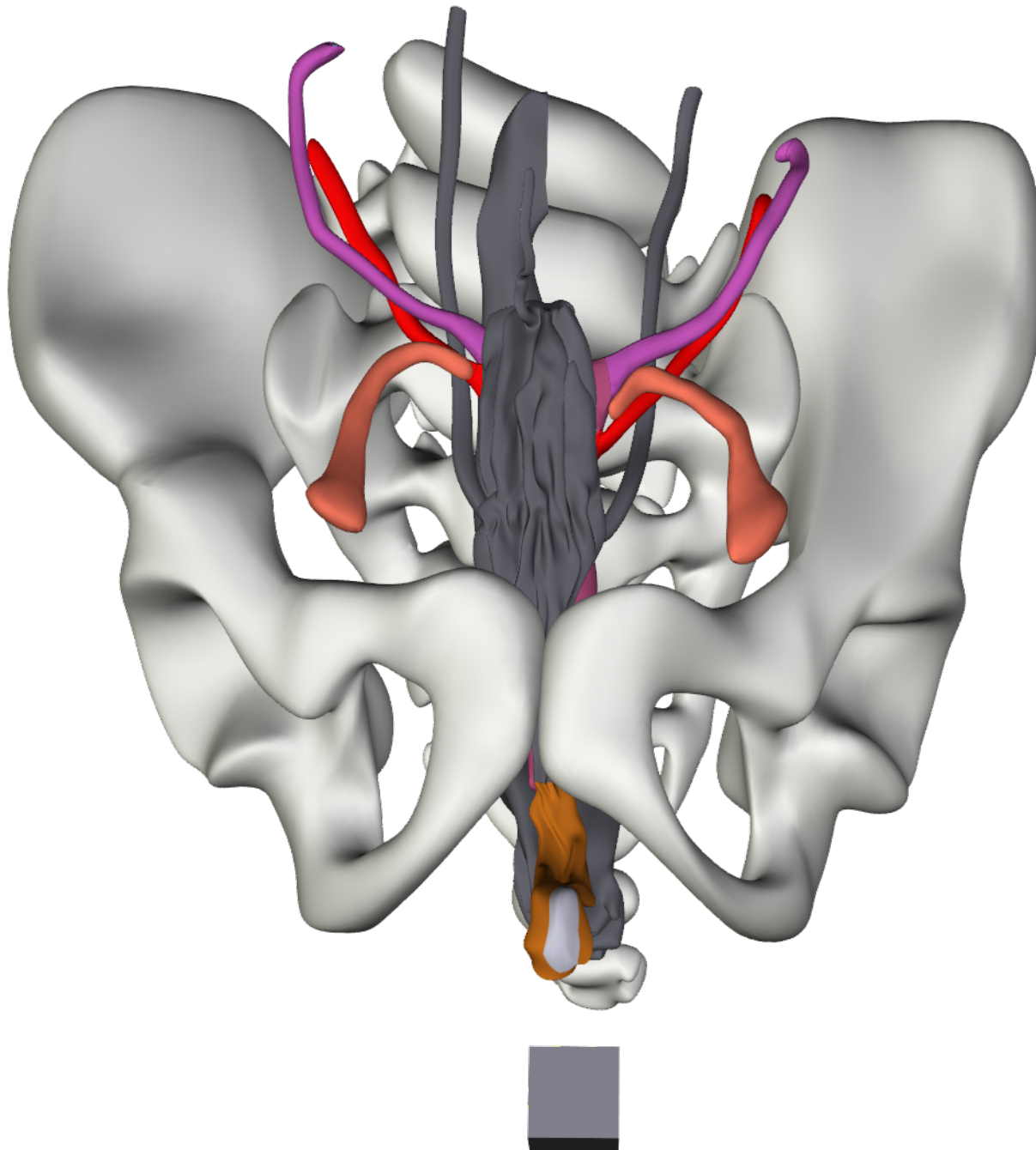

Supplement: Supplementary file 18 — Figure S18: ca70014‐sup‐0018‐FigureS18.pdf. Interactive 3D‐pdf of the urogenital region of a ~11‐week‐old human embryo (S1743). [file CA-39-92-s008.pdf]

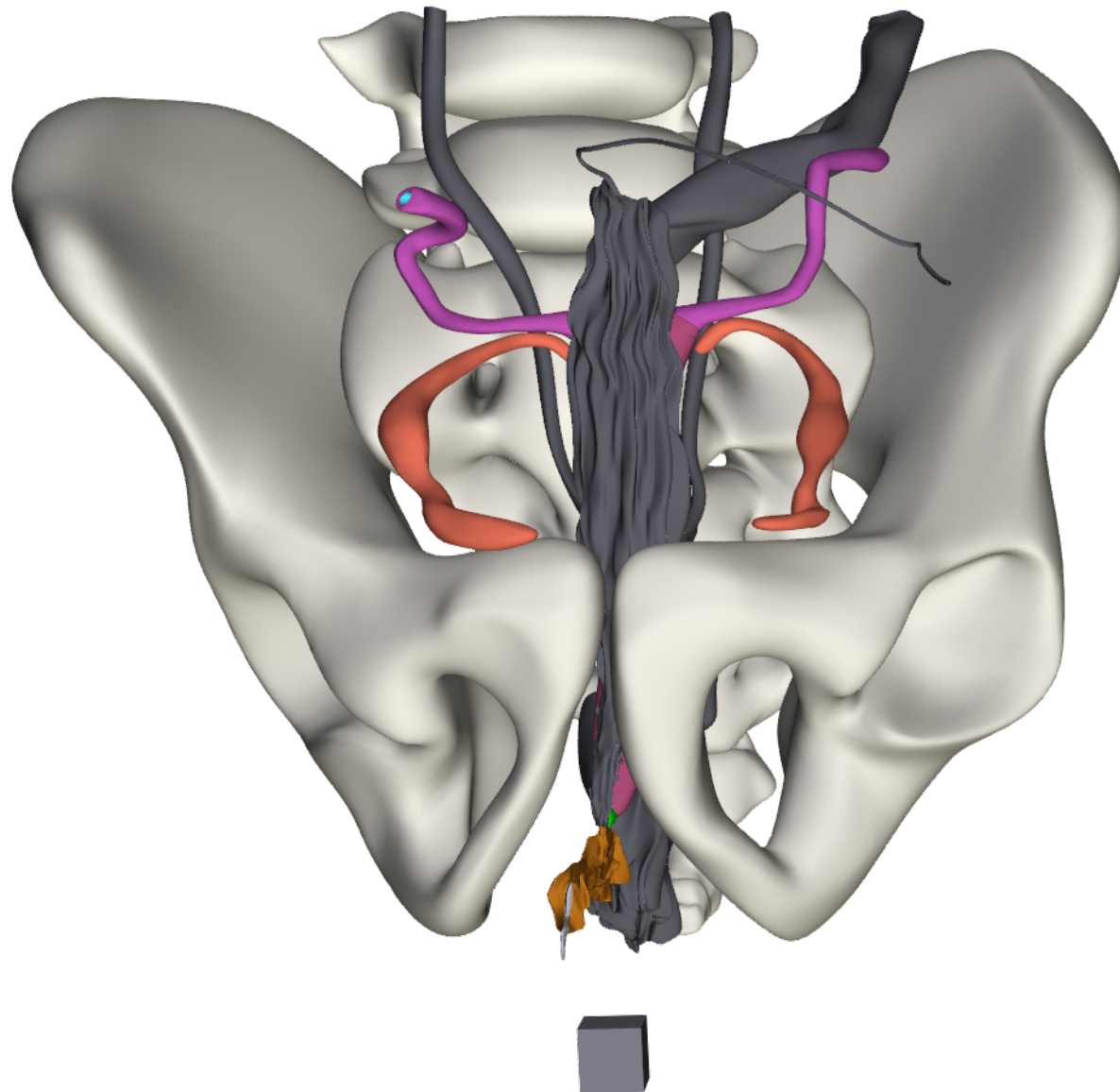

Supplement: Supplementary file 19 — Figure S19: ca70014‐sup‐0019‐FigureS19.pdf. Interactive 3D‐pdf of the urogenital region of a ~12‐week‐old human embryo (S2383). [file CA-39-92-s002.pdf]

13 Weeks (S2212)

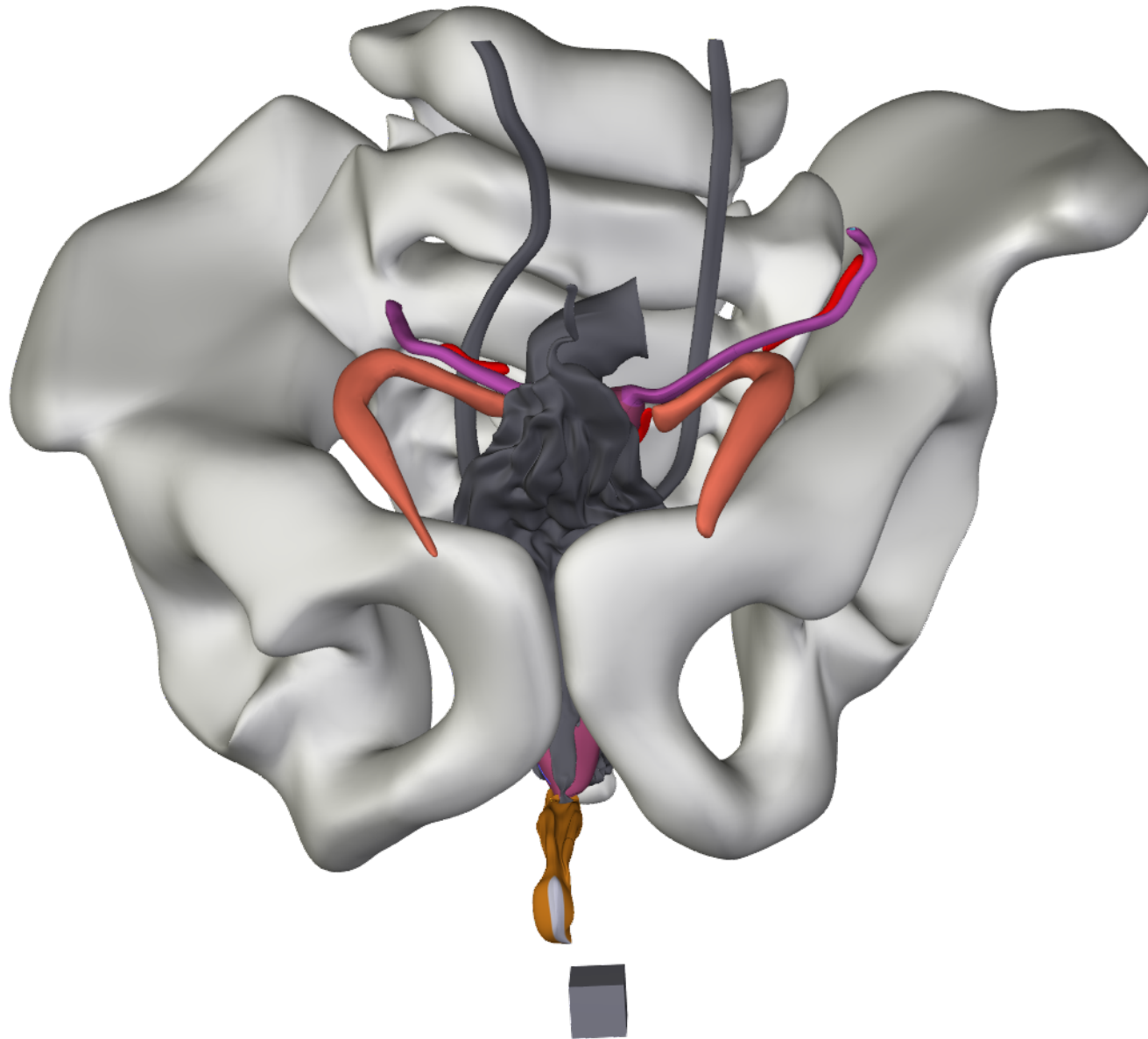

Supplement: Supplementary file 20 — Figure S20: ca70014‐sup‐0020‐FigureS20.pdf. Interactive 3D‐pdf of the urogenital region of a ~13‐week‐old human embryo (S2212). The cervix has a distinctive rugged surface. [file CA-39-92-s021.pdf]

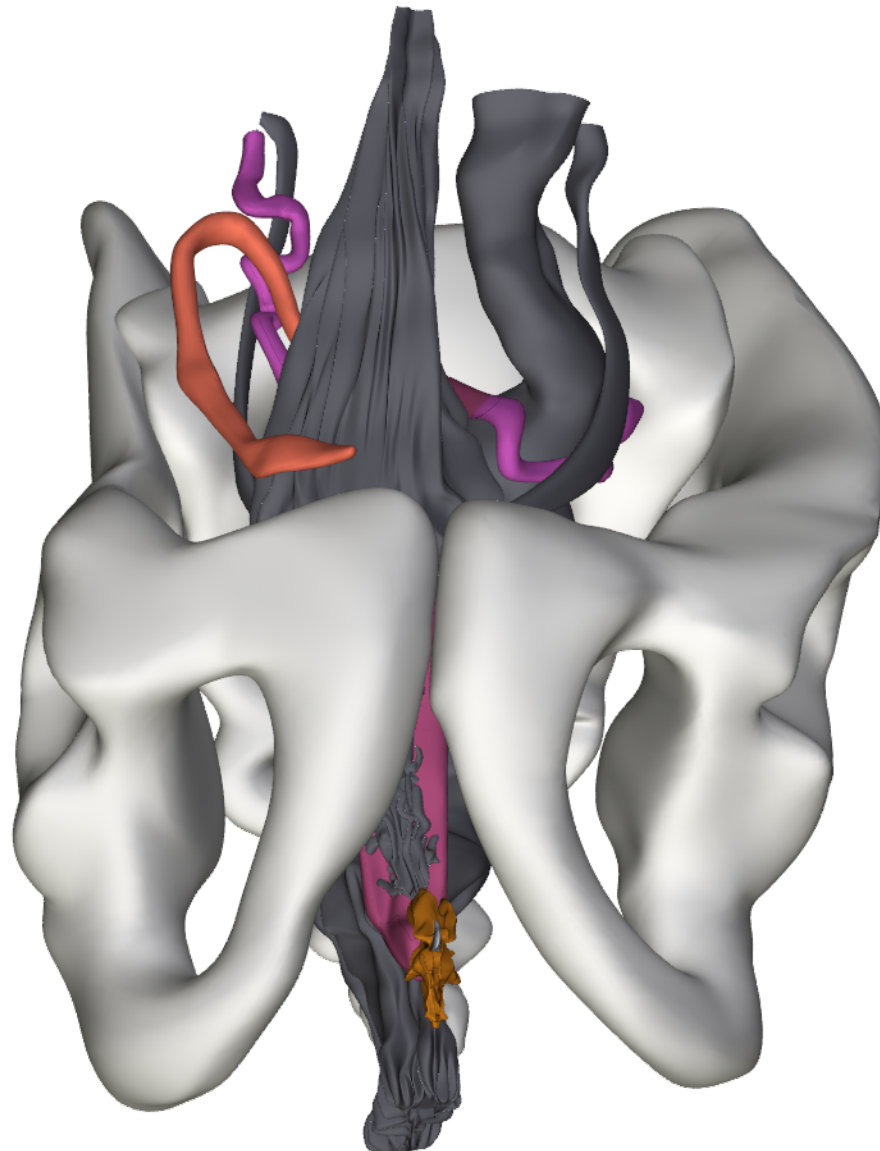

Supplement: Supplementary file 21 — Figure S21: ca70014‐sup‐0021‐FigureS21.pdf. Interactive 3D‐pdf of the urogenital region of a ~15‐week‐old human embryo (S2392). 30%–35% of the vagina is now positioned caudal from the pubococcygeal line. [file CA-39-92-s017.pdf]
